# Reciprocal activity of AgRP and POMC neurons governs coordinated control of feeding and metabolism

**DOI:** 10.1038/s42255-024-00987-z

**Published:** 2024-02-20

**Authors:** Alain J. De Solis, Almudena Del Río-Martín, Jan Radermacher, Weiyi Chen, Lukas Steuernagel, Corinna A. Bauder, Fynn R. Eggersmann, Donald A. Morgan, Anna-Lena Cremer, Michael Sué, Maximilian Germer, Christian Kukat, Stefan Vollmar, Heiko Backes, Kamal Rahmouni, Peter Kloppenburg, Jens C. Brüning

**Affiliations:** 1 Department of Neuronal Control of Metabolism, Max Planck Institute for Metabolism Research, Cologne, Germany; 2 Excellence Cluster on Cellular Stress Responses in Aging Associated Diseases (CECAD) and Center for Molecular Medicine Cologne (CMMC), University of Cologne, Cologne, Germany; 3 Policlinic for Endocrinology, Diabetes and Preventive Medicine (PEDP), University Hospital Cologne, Cologne, Germany; 4 Institute for Zoology, Biocenter, University of Cologne, Cologne, Germany; 5 Department of Neuroscience and Pharmacology, University of Iowa Carver College of Medicine, Iowa City, IA USA; 6 Multimodal Imaging of Brain Metabolism Group, Max Planck Institute for Metabolism Research, Cologne, Germany; 7 FACS & Imaging Core Facility, Max Planck Institute for Biology of Ageing, Cologne, Germany; 8 Fraternal Order of Eagles Diabetes Research Center, University of Iowa Carver College of Medicine, Iowa City, IA USA; 9 National Center for Diabetes Research (DZD), Neuherberg, Germany

**Keywords:** Hypothalamus, Neural circuits, Hypothalamus, Obesity

## Abstract

Agouti-related peptide (AgRP)-expressing and proopiomelanocortin (POMC)-expressing neurons reciprocally regulate food intake. Here, we combine non-interacting recombinases to simultaneously express functionally opposing chemogenetic receptors in AgRP and POMC neurons for comparing metabolic responses in male and female mice with simultaneous activation of AgRP and inhibition of POMC neurons with isolated activation of AgRP neurons or isolated inhibition of POMC neurons. We show that food intake is regulated by the additive effect of AgRP neuron activation and POMC neuron inhibition, while systemic insulin sensitivity and gluconeogenesis are differentially modulated by isolated-versus-simultaneous regulation of AgRP and POMC neurons. We identify a neurocircuit engaging Npy1R-expressing neurons in the paraventricular nucleus of the hypothalamus, where activated AgRP neurons and inhibited POMC neurons cooperate to promote food consumption and activate *Th*^+^ neurons in the nucleus tractus solitarii. Collectively, these results unveil how food intake is precisely regulated by the simultaneous bidirectional interplay between AgRP and POMC neurocircuits.

## Abstract (web summary)

De Solis and Del Río-Martín et al. investigate the reciprocal interplay between AgRP and POMC neurocircuits that governs the precise regulation of food intake and systemic metabolic homeostasis.

## Main

The central nervous system (CNS) regulates energy homeostasis in coordination with nutrient fluxes in peripheral tissues, constantly adjusting the balance between energy intake and expenditure to match the current internal state^1^. The hypothalamus comprises several defined areas with specific neuronal populations organized into neurocircuits that connect to multiple regions within the hypothalamus and other brain regions^2,3^. These neurocircuits integrate diverse internal and external signals, and, besides regulating food intake, they regulate numerous metabolic responses in peripheral organs^4^. Among others, two neuronal populations reside in the arcuate nucleus of the hypothalamus (ARC) with opposite effects on the regulation of metabolism: AgRP neurons, which are activated during states of energy deficit to promote foraging and food consumption^5–7^, and POMC neurons, which are activated in states of positive energy balance, which reduce food intake and increase energy expenditure (EE)^8^. Orexigenic, food intake-promoting signals, such as ghrelin, increase the neuronal activity of AgRP neurons, which release the inhibitory neurotransmitter GABA (gamma-aminobutyric acid) together with neuropeptide Y (NPY) and AgRP and subsequently inhibit downstream neurons^8–10^. On the other hand, anorexigenic, food intake-suppressing hormones, such as leptin, GLP-1 and insulin, activate POMC neurons while inhibiting AgRP neurons. POMC neurons release several peptides derived from the post-translational processing of precursor neuropeptide POMC^11^. These neuropeptides act to inhibit or activate downstream neurons in intrahypothalamic and extrahypothalamic areas connected to the extensive AgRP and POMC axonal projections^12^. Through these downstream neurocircuits, AgRP and POMC neurons exert their effects over feeding behaviour and several other metabolic functions, including insulin sensitivity, control of hepatic gluconeogenesis and the lipolysis in adipose tissue depots^13–15^. Nevertheless, the downstream neurocircuits, responsible for integrating signals from both AgRP and POMC populations either individually or simultaneously, have not been fully characterized yet.

In addition to their homeostatic hormonal regulation, AgRP and POMC neurons receive extensive neuronal input from several brain areas, and activity levels of AgRP and POMC neurons are rapidly modulated via top–down neuronal input control^16^. Here, sensory food perception-dependent POMC neuron activation increases sympathetic nerve activity (SNA) in the liver, which primes this organ for nutrient partitioning and substrate utilization^17^. In contrast, AgRP neuronal activation reduces SNA in white adipose tissue and brown adipose tissue (BAT) and cardiovascular tissues^13,15,18^.

Tremendous advances in our current understanding of the pleiotropic physiological responses governed by AgRP and POMC neurons, and their anatomical and functional neurocircuit organization have resulted from recent developments of modern neuroscience techniques. These approaches, largely based on Cre/loxP-mediated recombination, have also enhanced our understanding about the role of AgRP and POMC neurons in metabolic control. However, these studies so far have only modulated one of these cell populations at a time, while naturally both neuronal populations are simultaneously regulated in opposite directions^16^. In the present study, we aimed to dissect whether the reciprocal interplay between AgRP and POMC neurocircuits participates in the regulation of metabolic homeostasis by cooperative, synergistic or maybe even antagonistic actions.

Here, we designed a transgenic approach that combines Dre/rox-dependent and Cre/loxP-dependent recombination^19,20^ to express functionally opposite DREADD receptors in either cell population to mimic the physiologically occurring simultaneous bidirectional regulation of AgRP and POMC neurons. These experiments revealed that food consumption is regulated by the additive effects of combined reciprocal chemogenetic modulation of AgRP and POMC neurons. We explored which CNS areas integrate the simultaneous reciprocal signals from AgRP and POMC neurons. Analysis of key neuronal populations in the paraventricular nucleus of the hypothalamus (PVH) followed by an unbiased whole-brain analysis identified a downstream neurocircuitry composed of Npy1R^PVH^ neurons. This PVH subpopulation integrates neuronal input from both AgRP and POMC neurons, participates in the regulation of feeding behaviour and activates Th^NTS^ neurons. However, we also found that several metabolic responses during the feeding/fasting transition are differentially controlled by the antagonistic interplay between AgRP and POMC neurocircuits compared to their isolated effects. Altogether, these results demonstrate how the interplay between AgRP and POMC neurocircuits regulates food intake and the metabolic response of peripheral tissues during feeding-state transitions and identified distinct and overlapping functions governed by AgRP and POMC neurocircuits in the regulation of metabolic homeostasis.

## Results

### Simultaneous expression of reciprocal DREADDs in AgRP and POMC neurons

**Fig. 1:**
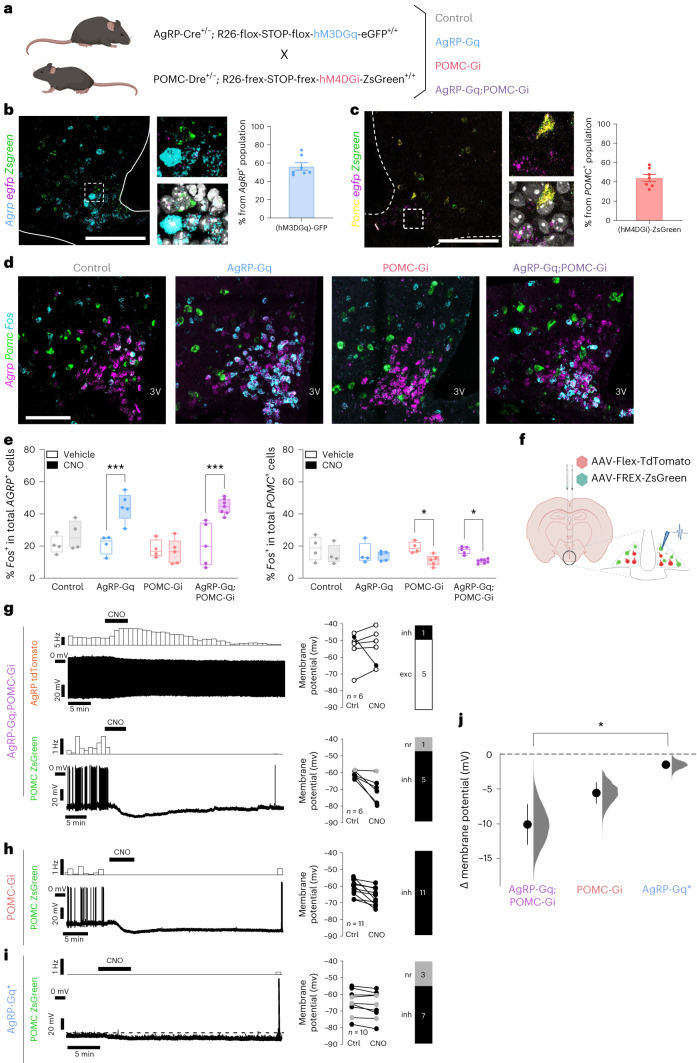
Cre and Dre recombinases drive specific expression of opposite chemogenetic receptors in AgRP and POMC neurons. **a**, Breeding strategy and experimental groups. **b**, Representative images and quantification of Cre-mediated hM3DGq-eGFP expression in AgRP neurons (*n* = 7 mice) **c**, Representative image and quantification of Dre-mediated hM4DGi-ZsGreen expression in POMC neurons (*n* = 7 mice). **d**, Representative images showing *Fos* (cyan) colocalization with *AgRP*^*+*^ (magenta) and *POMC*^*+*^ (green) neurons in experimental groups treated with CNO for 1 h. **e**, Quantification of percentage of *AgRP*^*+*^ and *POMC*^*+*^ neurons colocalized with *Fos* mRNA 1 h after vehicle or CNO injection. *AgRP*^+^ neurons: ***P = 0.0007 AgRP-Gq, ***P = 0.0001 AgRP-Gq;POMC-Gi, vehicle versus CNO; *POMC*^+^ neurons: *P = 0.0375 POMC-Gi, *P = 0.473 AgRP-Gq;POMC-Gi, vehicle versus CNO. Data are presented as the mean ± s.e.m. of the percentage of AgRP or POMC neurons in one ARC hemisection (*n* = 4 and 4 control mice; 4 and 5 AgRP-Gq mice; 4 and 5 POMC-Gi mice; 5 and 7 AgRP-Gq;POMC-Gi mice, vehicle versus CNO, respectively, for each group). Statistical significance was determined by two-way analysis of variance (ANOVA) followed by Bonferroni’s test. Box plots indicate the median ± minimum/maximum values and include data points of individual mice. Scale bar, 100 µm. **f**, Scheme of experimental design. **g**–**i**, Perforated patch-clamp recordings and rate-histograms displaying the effects of 3 µM CNO on POMC-ZsGreen^+^ and AgRP-tdTomato^+^ neurons. Bar graphs represent the proportion of neurons classified as CNO responders at the individual-cell level (Methods): excited (exc, white), not responsive (nr, grey) and inhibited (inh, black). All recordings were performed in the presence of glutamate and GABA receptor blockers, except **i**. **g**, Recordings from AgRP-tdTomato^+^ neurons (upper) and from POMC-ZsGreen^+^ neurons (lower) from AgRP-Gq:POMC-Gi mice (*n* = 6 cells). **h**, Recordings from POMC-ZsGreen^+^ neurons from POMC-Gi mice (*n* = 11 cells). **i**, Recording from POMC-ZsGreen^+^ neurons from AgRP-Gq* mice (*n* = 10 cells) in the absence of synaptic blockers. The dashed line serves to emphasize the effect. **j**, Summarized effects of 3 µM CNO-mediated DREADD activation in POMC neurons. Data are displayed as the mean ± s.e.m. of changes in membrane potential calculated from all recordings shown in **g**–**i**. All population responses measured in the respective experimental groups were statistically significant AgRP-Gq:POMC-Gi (−10.1 ± 2.9 mV, *n* = 6, *P* = 0.03, Wilcoxon matched-pairs signed-rank test), POMC-Gi (−5.6 ± 1.5 mV, *n* = 11, *P* = 0.004, two-tailed paired *t*-test) and AgRP-Gq (−1.6 ± 0.6 mV, *n* = 10, *P* = 0.03, two-tailed paired *t*-test). Comparison of the effect sizes between groups showed a significant difference in POMC neuron hyperpolarization between AgRP-Gq:POMC-Gi and AgRP-Gq* mice (*P* = 0.01; Kruskal–Wallis test with Dunn’s multiple-comparison test). Based on the observed data, the filled grey curves indicate the resampled distribution (5,000 bootstrap samples). Figure 1a,f created with BioRender.com. Source data

To address how simultaneous bidirectional regulation of AgRP and POMC neurons controls metabolism, we used non-interacting recombinases to simultaneously express excitatory and inhibitory DREADDs in AgRP and POMC neurons. To this end, we crossed AgRP-IRES-Cre transgenic mice with mice allowing for Cre-dependent expression of the excitatory hM3DGq receptor (CAG-flox-Stop-flox-hM3Gq-eGFP) as well as mice expressing the alternative recombinase Dre in POMC neurons^21^ with mice allowing for Dre-dependent expression of the inhibitory hM4DGi receptor (CAG-rox-stop-rox-hM4DGi-ZsGreen). Further intercrosses between these two lines yielded four experimental groups of animals, that is, mice with isolated clozapine *N*-oxide (CNO)-dependent activation of AgRP neurons (AgRP-Gq), mice with CNO-dependent inhibition of POMC neurons (POMC-Gi), and those with simultaneous activation of AgRP and inhibition of POMC neurons (AgRP-Gq;POMC-Gi), while for controls in the absence of both recombinases, neither expressed hM3DGq nor hM4DGi (Fig. 1a).

**Extended Data Fig. 1:**
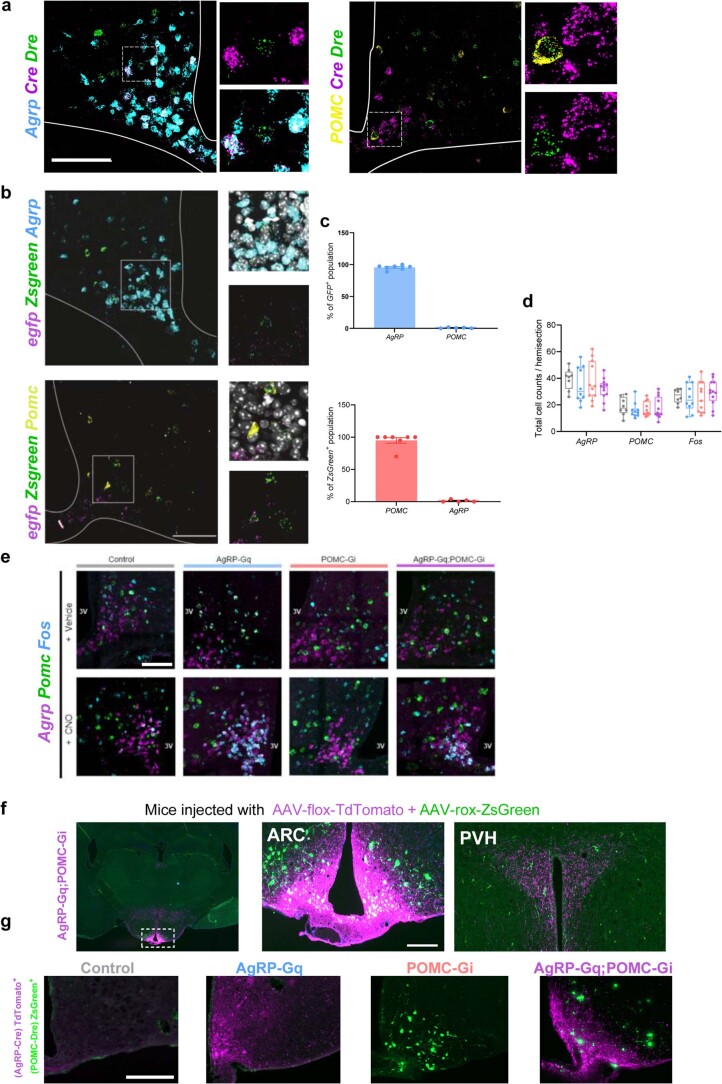
Selective expression of Cre- and Dre- recombinases in AgRP and POMC neurons. **a**, Representative images of exclusive localization of *Cre* recombinase (magenta) on *AgRP*^*+*^ neurons (cyan) and *Dre* recombinase (green) on *POMC*^*+*^ neurons (yellow) (*n* = 4 mice). **b**, Representative images showing the expression of *egfp* (magenta) and *Zsgreen* (green) mRNA, attached markers of DREADDs hM3DGq and hM4DGi, respectively, together with *Fos* mRNA (cyan) in sections from CNO-treated AgRP-Gq;POMC-Gi mice (*n* = 7 mice/group). **c**, Quantification of the percentage of AgRP and POMC neurons that co-express *egfp* or *Zsgreen* markers, (*n* = 3 AgRP-Gq and 4 AgRP-Gq;POMC-Gi mice for upper panel, 3 POMC-Gi and 4 AgRP-Gq;POMC-Gi mice for lower panel). **d**, Quantification of total cell counts expressing *Agrp*, *P* or *Fos* mRNA from experimental groups in basal conditions. Boxplots indicate median ±min/max and include data points of individual mice. (*n* = 8 control, 10 AgRP-Gq, 11 POMC-Gi and 12 AgRP-Gq;POMC-Gi mice). **e**, Representative images from coronal ARC sections showing the colocalization of *Agrp* (magenta) and *POMC* (green) neurons with *Fos* (cyan) after experimental mice were injected with vehicle or CNO. **f**, Representative images showing the distribution AgRP-Tomato^+^ and POMC-ZsGreen^+^ axonal projections in the hypothalamus, ARC and PVH areas from AgRP-Gq;POMC-Gi mice bilaterally injected with AAV-flox-tdTomato + AAV-rox-Zsgreen. **g**, Representative images showing the selective recombination and expression of fluorescent proteins by AgRP and POMC neurons. Mice from all experimental groups were injected bilaterally with a mix of AAV-flox-tdTomato + AAV-frex-ZsGreen (*n* = 2 AgRP-Gq;POMC-Gi mice). Data represent the mean ± s.e.m for each group and treatment. Source data and further details of statistical analyses are provided as a Source Data file. Scale bar = 100 µm. Source data

**Extended Data Fig. 2:**
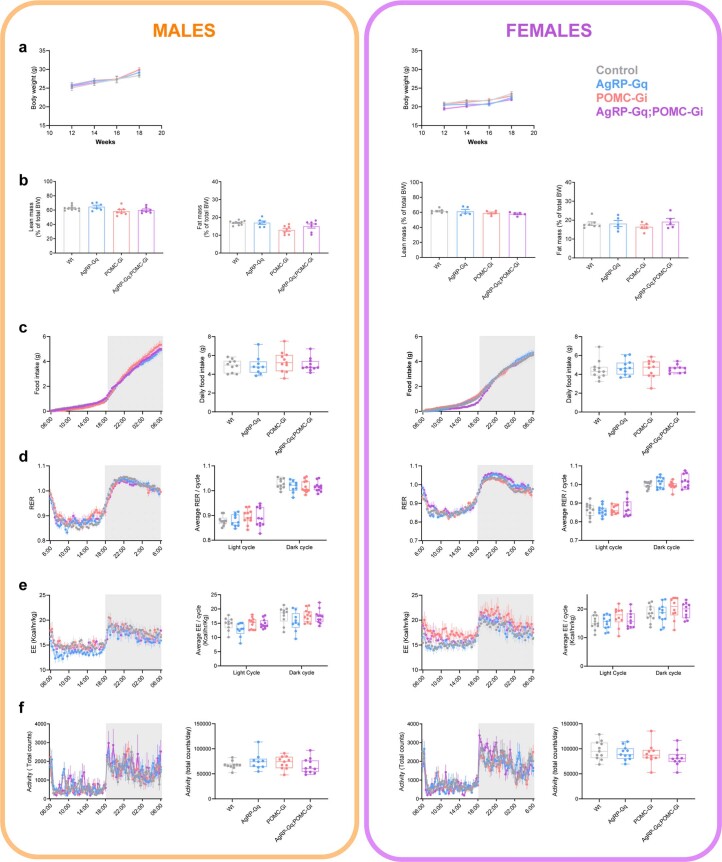
Metabolic phenotyping of experimental mouse lines in basal conditions. Data from males (**left**) and females (**right**) from the four experimental groups showing: **a**, Body weight curves between 12 and 18 weeks of age. **b**, Body weight composition. **c**, Daily basal food intake, in cumulative curve and daily average. **d**, Respiratory coefficient (RER) curves and averages for light and dark cycles. **e**, Energy expenditure (EE) curves and averages for the light and dark cycles **f**, Total activity counts across 24h and average of daily activity. Data is presented as mean ± s.e.m from the biological replicates in each group (control, AgRP-Gq, POMC-Gi and AgRP-Gq;POMC-Gi, respectively) in **a** (*n* = 18, 16, 19, 20 male mice and 16, 20, 20, 18 female mice), **b** (*n* = 10, 6, 7, 7 male mice and 7, 5, 5, 5 female mice), **c–f** (*n* = 10, 9, 11, 11 male mice and 11, 11, 10, 10 female mice). Boxplots indicate median ± min/max and include data points of individual mice entering the analysis. Statistical test: Mixed-effects model in **a** and **d-**, **e-left panels**, one-way ANOVA in **b** and **c-**, **f-right panel**, and two-way ANOVA in **c-**, **f-left panels** and, **d**,**-**,**e-right panel**. Source data and further details of statistical analyses are provided as a Source Data file. Source data

To validate our transgenic model, we first localized the mRNA expression of either recombinase by RNAscope-based fluorescence in situ hybridization (FISH). *Cre* mRNA was selectively detected in AgRP neurons and *Dre* mRNA was only detected in POMC-expressing neurons, with no overlap in the expression of either recombinase (Extended Data Fig. 1a). The quantification of DREADDs expression showed that 56.32% ± 4.0% of all AgRP neurons expressed *gfp* mRNA along with hM3DGq (Fig. 1b), whereas 44.2% ± 3.6% of all POMC neurons expressed *Zsgreen* mRNA as a read-out for hM4DGi receptor expression (Fig. 1c and Extended Data Fig. 1b,c). Moreover, we did not detect significant changes in ARC cell numbers or any metabolic alterations in male and female transgenic mice of the four experimental groups in the absence of CNO treatment (Extended Data Fig. 1d and Extended Data Fig. 2).

### Functional validation of AgRP-Gq, POMC-Gi and AgRP-Gq;POMC-Gi mice

To validate the effect of functionally opposing DREADDs expressed in AgRP or POMC neurons, we first quantified the percentage of AgRP and POMC neurons that co-express *Fos* mRNA by FISH. Ad libitum-fed mice were treated at the beginning of the light cycle with either vehicle or CNO for 1 h without further access to food (Fig. 1d and Extended Data Fig. 1e). In vehicle-treated mice from all four experimental groups, we identified an average of 20.24% ± 0.7% of *AgRP*-expressing neurons that colocalize with *Fos* mRNA, and an average of 17.5% ± 1.0% of all endogenously *POMC*^*+*^-expressing neurons that co-expressed *Fos*. In control mice treated with vehicle or CNO, we did not observe changes in the number of *Fos*^+^ in *AgRP*^*+*^ or *POMC*^*+*^ neurons, indicating that CNO injection had no effects on these ARC neurons in the absence of DREADD expression (Fig. 1e). Following CNO treatment in AgRP-Gq and AgRP-Gq;POMC-Gi mice, ≈45% of *AgRP*^+^ neurons colocalized with *Fos*, doubling the percentage observed in vehicle-treated mice (20.9% ± 6% versus 44% ± 9%, in AgRP-Gq mice and 21.0% ± 6.0% versus 44.5% ± 2.2% in AgRP-Gq;POMC-Gi mice, vehicle versus CNO; Fig. 1d,e). In POMC-Gi and AgRP-Gq;POMC-Gi mice, CNO treatment induced a reduction in the percentage of *Fos*^+^, *POMC*^+^ neurons (19.6% ± 3% versus 12.8% ± 4% in POMC-Gi mice and 16.7% ± 1.2% versus 11.7% ± 1.1% in AgRP-Gq;POMC-Gi mice, vehicle versus CNO; Fig. 1e). Interestingly, we did not observe a significant reduction of *Fos*^+^, *POMC*^+^ neurons in AgRP-Gq mice treated with CNO (15.1% ± 6.0% versus 13.3% ± 3.0 %, vehicle versus CNO; Fig. 1e).

### Electrophysiological characterization of reciprocal DREADD actions in AgRP and POMC neurons

Next, we assessed the neuronal responses of AgRP and POMC neurons following CNO application using perforated patch-clamp recordings in brain slices of adult mice. To facilitate visualization, AgRP and POMC neurons were genetically marked via stereotaxic injection of Cre-dependent or Dre-dependent adeno-associated virus (AAV) constructs containing AAV-CAG-flex-tdTomato or AAV-CAG-Frex-ZsGreen into the ARC (Fig. 1f). tdTomato protein was expressed only in AgRP neurons, while the ZsGreen signal was restricted to POMC neurons (Extended Data Fig. 1f,g). Furthermore, the injection of both AAVs into the ARC of AgRP-Gq;POMC-Gi mice not only confirmed the selective differential targeting of AgRP and POMC neurons but also allowed the visualization of the downstream areas receiving concurrent axonal projections from AgRP and POMC neurons (Supplementary Figs. 1 and 2). As previously published^12^, these analyses revealed a wide distribution of areas that receive innervation from AgRP and POMC neurons indicating that several downstream areas may integrate neuronal input from both neurocircuits simultaneously.

During electrophysiological recordings, CNO was bath-applied at a concentration of 3 µM and glutamatergic and GABAergic synaptic inputs were pharmacologically blocked unless otherwise stated (Fig. 1g–i). In AgRP-Gq;POMC-Gi mice, in 83% (5 of 6) of tdTomato-labelled AgRP neurons, CNO application evoked a clear excitation (Fig. 1g), while 83% (5 of 6) of recorded ZsGreen-labelled POMC neurons showed a significant CNO-dependent inhibition (Fig. 1g). In POMC-Gi mice, ZsGreen-labelled POMC neurons showed robust inhibition in 100% (11 of 11) of recorded neurons (Fig. 1h). Finally, in AgRP-Gq mice, CNO application indirectly induced hyperpolarization in 70% (7 of 10) of ZsGreen-labelled POMC neurons via activation of GABAergic AgRP-Gq neurons when the synaptic transmission was not pharmacologically blocked (Fig. 1i). Comparing the mean effects of all recordings of the respective experimental groups showed that the combined direct and indirect inhibition of ZsGreen-labelled POMC neurons in AgRP-Gq;POMC-Gi mice was significantly greater than the indirect inhibition of POMC neurons in AgRP-Gq mice alone (Fig. 1j). Collectively, these results confirm the specific expression and functionality of opposite chemogenetic receptors upon a single CNO application.

### The interplay between AgRP and POMC neurons cooperates to increase food consumption

**Fig. 2:**
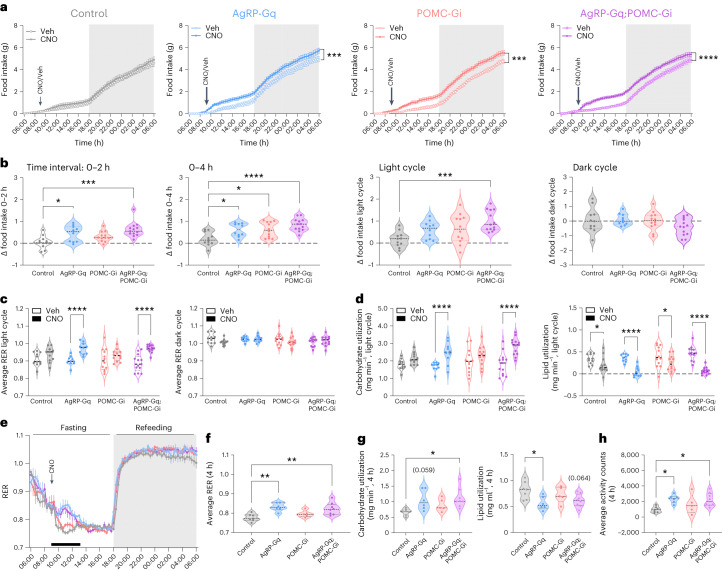
Coordinated effect in the regulation of food intake from the interaction of AgRP and POMC neurocircuits. **a**, Cumulative food intake in male mice treated with vehicle and CNO in crossover experimental design. Dark cycle is indicated by the grey rectangle. ***P < 0.004 AgRP-Gq and ***P = 0.009 POMC-Gi, ****P < 0.0001 AgRP-Gq;POMC-Gi, vehicle versus CNO. **b**, Quantification of delta food intake (∆ = CNO − vehicle) during the indicated time intervals. Control versus AgRP-Gq: *P = 0.0292 (2 h) and *P = 0.0193 (4 h); control versus POMC-Gi: *P = 0.012 (4 h); control versus AgRP-Gq;POMC-Gi: ***P = 0.0003 (2 h), ****P < 0.0001 (4 h) and ***P = 0.0009 (light cycle) **c**, Average RER during light and dark cycles in male mice treated with vehicle or CNO. ****P < 0.0001, vehicle versus CNO. **d**, Quantification of carbohydrate and lipid utilization during light-cycle period by male mice treated with CNO and vehicle, calculated as: *P = 0.0359 for control, *P = 0.0121 for POMC-Gi, ****P < 0.001 for AgRP-Gq and AgRP-Gq;POMC-Gi groups, vehicle versus CNO. **e**, RER curves in male mice treated with CNO without access to food during the light cycle, following by refeeding at the beginning of the dark cycle. Black bar indicates the 4-h period after CNO injection. **f**, Average RER during the 4-h period. **P = 0.0021 and **P = 0.0067 for control versus AgRP-Gq and AgRP-Gq;POMC-Gi, respectively. **g**, Quantification of carbohydrate and lipid usage during the 4-h fasting period. Carbohydrates: *P = 0.0137 control versus AgRP-Gq;POMC-Gi; lipids: *P = 0.0222 control versus AgRP-Gq. **h**, Total activity counts during the 4-h period. *P = 0.026 and *P = 0.0259 for control versus AgRP-Gq and AgRP-Gq;POMC-Gi, respectively. Data represent the mean ± s.e.m. of the biological replicates for each group: **a**–**d**: *n* = 11 control, 10 AgRP-Gq, 12 POMC-Gi and 13 AgRP-Gq;POMC-Gi male mice; **e**–**j**: *n* = 7 control, 6 AgRP-Gq, 5 POMC-Gi and 8 AgRP-Gq;POMC-Gi male mice. Statistical significance was determined by two-way ANOVA followed by Bonferroni test for **a** and **c**–**e**; and one-way ANOVA followed by Tukey’s multiple-comparison test for **b** and **f**–**h**. Data from female mice are displayed in Extended Data Fig. 3. Source data

**Extended Data Fig. 3:**
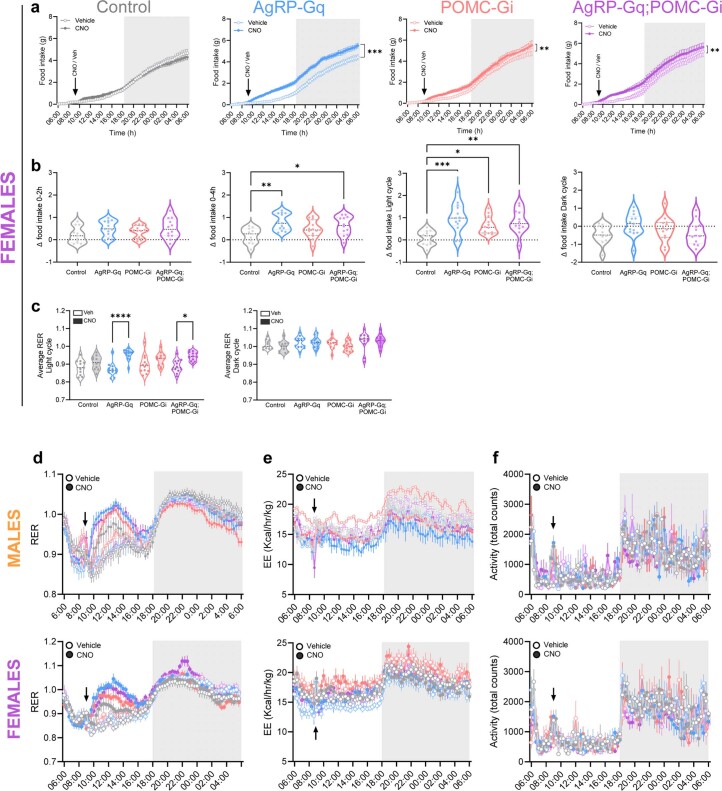
The cooperative regulation of food intake from the interplay of AgRP and POMC neurocircuits is also present in female mice. **a**, Cumulative food intake for 24h in female mice treated with vehicle and CNO in a cross-over experimental design ***P = 0.0002 AgRP-Gq, **P = 0.0042 POMC-Gi and **P = 0.0031 AgRP-Gq;POMC-Gi, Veh vs. CNO, respectively. **b**, Quantification of delta food intake (∆ = CNO - Vehicle) during the indicated time intervals after CNO or vehicle injection in female mice 4h: **P = 0.0019 AgRP-Gq, *P = 0.0375 AgRP-Gq;POMC-Gi; Light cycle: ***P = 0.00008 AgRP-Gq, *P = 0.0269 POMC-Gi, **P = 0.0024 AgRP-Gq;POMC-Gi. **c**, Average of respiratory exchange ratio (RER) during light and dark cycles in female mice treated with vehicle or CNO.***P = 0.0002 AgRP-Gq, *P = 0.0113 AgRP-Gq;POMC-Gi, Veh vs. CNO. **d**, Curves depicting the changes in RER after injection of vehicle or CNO during the light cycle in male (**upper**) or female (**lower**) mice. **e**, Curves showing no differences in EE in experimental male and female mice treated with CNO or vehicle. **f**, Curves showing no changes between experimental groups in the total activity curves of male and female mice after CNO or vehicle injection. Data showed the mean ± s.e.m. for each group, with *n =* 11 control, 13 AgRP-Gq, 12 POMC-Gi and 12 AgRP-Gq;POMC-Gi female mice in **a – c** and **lower panels d – f**; and *n* = 11 control, 10 AgRP-Gq, 12 POMC-Gi, 13 AgRP-Gi;POMC-Gq male mice in **upper panel d – f**. Statistical test: 1-way ANOVA followed by Tukey multiple comparison test for **b**, **c**; 2-way ANOVA followed by Bonferroni′s multiple comparisons test for **a**, and 2-way ANOVA Mixed effects model for **d – f**. Source data and further details of statistical analyses are provided as a Source Data file. Source data

We next investigated how isolated or simultaneous bidirectional regulation of AgRP and POMC neurons affects feeding behaviour. Male and female mice of the four experimental groups were placed into metabolic chambers and were injected with vehicle or CNO at the beginning of the light cycle. CNO injection in control mice did not cause any change in food intake, EE or activity patterns compared to vehicle-treated mice (Fig. 2a,b and Extended Data Fig. 3a–f). In male mice, CNO-induced AgRP activation in AgRP-Gq mice resulted in a rapid increase in food intake during the first 2 h following CNO injection, and this response was sustained for 6 h, although it was no longer significantly different after 8 h (Fig. 2a,b), consistent with several previous reports^18,22,23^. In contrast, male POMC-Gi mice increased their food consumption after 4 h following CNO injection (Fig. 2b), and this effect was no longer present at the end of the light cycle. Finally, we analysed food intake in male AgRP-Gq;POMC-Gi mice, which showed a rapid increase in food consumption within 2 h following CNO injection. Interestingly, in this group, food intake remained higher throughout the entire light cycle, in contrast to the effects of isolated AgRP activation or isolated POMC inhibition (Fig. 2a,b). In female mice (Extended Data Fig. 3a–c), these results were also observed, although they were delayed, observing the significative increase in food intake driven by AgRP activation at 4 h, and at 8 h in POMC-Gi female mice (Extended Data Fig. 3a,b). However, the cooperative effect on food intake in AgRP-Gq;POMC-Gi mice was not fully conserved because all female mice rapidly increased their food intake at the beginning of the dark cycle.

### Fasting-induced alterations in nutrient flux are primarily controlled by AgRP neurons

We also compared the effects of isolated-versus-combined regulation of AgRP and POMC neurons on the respiratory exchange ratio (RER) and EE. Indirect calorimetry measurements in male and female mice showed an increase in RER during the light cycle that parallelled the increase in food intake of CNO-injected AgRP-Gq and AgRP-Gq;POMC-Gi mice (Fig. 2c and Extended Data Fig. 3c), without further alterations in EE (Extended Data Fig. 3e). However, CNO-injected POMC-Gi mice exhibited no significant changes in RER during the light-cycle phase, despite their increase in food consumption during the same period (Fig. 2b,c and Extended Data Fig. 3b,c). We next analysed the CNO-induced changes in carbohydrate and lipid utilization in the four different groups of male mice. Chemogenetic activation of AgRP neurons in male AgRP-Gq and AgRP-Gq;POMC-Gi groups resulted in higher carbohydrate utilization and lower lipid usage during the light cycle following CNO injection (Fig. 2d). However, these changes in substrate utilization were not present in POMC-Gi mice, despite that they also increased food intake during the light cycle comparable to AgRP-Gq mice (Fig. 2b,d).

We further investigated the effect of CNO injection in the different groups of male mice in the absence of food supply. As previously described, chemogenetic activation of AgRP neurons produced a short-term elevation of the RER over a period of 4 h (Fig. 2e,f) and a shift towards the use of carbohydrates as primary energy source, together with reduced lipid catabolism (Fig. 2g). On the other hand, control and POMC-Gi mice exhibited a continuous decline in RER during the same period (Fig. 2e,f), suggesting a predominant use of lipids during prolonged fasting (Fig. 2g). The acute RER elevation induced by AgRP activation was accompanied by an increased activity pattern, indicative of foraging behaviour, in AgRP-Gq and AgRP-Gq;POMC-Gi mice (Fig. 2h). These results indicate that AgRP neurons control overall nutrient flux and goal-oriented behaviours without depending on concomitant POMC neuron inhibition.

**Extended Data Fig. 4:**
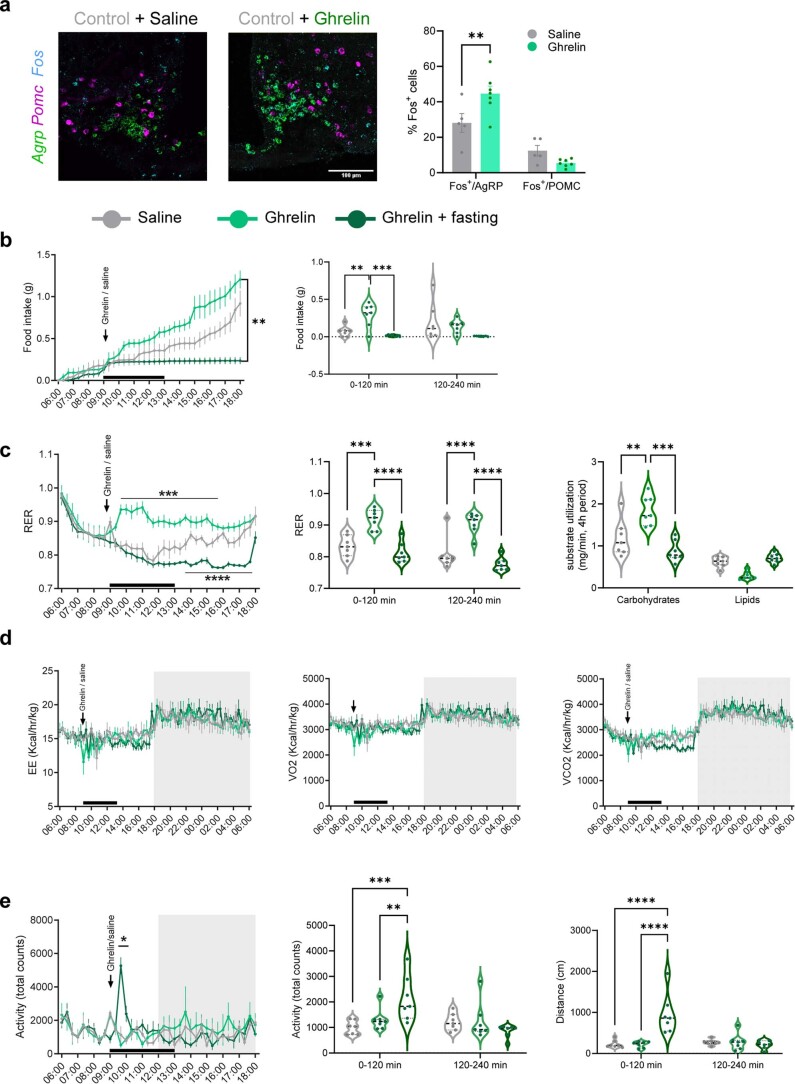
Metabolic effects of ghrelin injection in male control mice. **a**, Representative images showing the colocalization of *Fos* mRNA in *AgRP*^*+*^ and *POMC+* neurons from control mice treated with saline or ghrelin (**left panel**) for 1h; and quantification of the percentage of activated (*Fos*^+^) *AgRP*^*+*^ and *POMC*^*+*^ neurons after ghrelin injection (**right panel**) * P = 0.0081. **b**, **left panel:** Cumulative food intake of male control mice treated with saline or ghrelin in the presence or absence of food. ** P = 0.005; **right panel**: Total food intake during time intervals. **P = 0.0047 ***P = 0.0008. **c**, l**eft panel**: Respiratory coefficient during light cycle ***P = 0.0007, ****P < 0.0001, **central panel:** average of RER during indicated time intervals *** P = 0.0001, ****P < 0.0001, and **right panel**: average nutrient flux during the 4h after ghrelin injection **P = 0.0033, *** P = 0.0001. **d**, Average Energy expenditure (EE), VO_2_, and VCO^2^ measurements during 24h. **e**, **left panel**: Activity counts during 24h measurement. *P = 0.0144 fast vs. saline, **central panel:** average activity counts ***P = 0.0003, **P = .0035, and **right panel:** distance traveled during the time intervals. ****P < 0.0001. Data are indicated as mean ± s.e.m. of cell counts per ARC hemisection *n* = 5 saline- and 7 ghrelin-injected male control mice in **a**, and as mean ± s.e.m from *n* = 7 male control mice in **b – e**. Statistical analysis: 2-way ANOVA followed by Bonferroni’s multiple comparison in **a**, and Tukey’s multiple comparisons in **b – e**. Source data and further details of statistical analyses are provided as a Source Data file. Source data

These adaptations were consistent with the metabolic changes caused by ghrelin, which acts predominantly via the activation of AgRP neurons^24^. Ghrelin injection in male control mice caused the activation of AgRP neurons to a similar degree as observed after chemogenetic AgRP neuron activation (44.6% ± 4.2% versus 28.10% ± 5.2% of *Fos*^+^, *AgRP*^+^ neurons, ghrelin versus vehicle) without causing a significant decrease in POMC neuronal activity (Extended Data Fig. 4a). Ghrelin injection caused a rapid increase in food intake during the first 2 h following injection and increased RER and carbohydrate usage without altering the EE (Extended Data Fig. 4b–d). However, ghrelin injection in the absence of food caused a clear increase in physical activity and distance travelled during the first 120 min, indicative of foraging behaviours, but no changes in EE (Extended Data Fig. 4d,e). Altogether, these results suggest that foraging behaviours and nutrient flux are controlled mainly by the activation of AgRP neurons.

### Identification of downstream neuronal circuits that integrate simultaneous signals from AgRP and POMC neurons

**Fig. 3:**
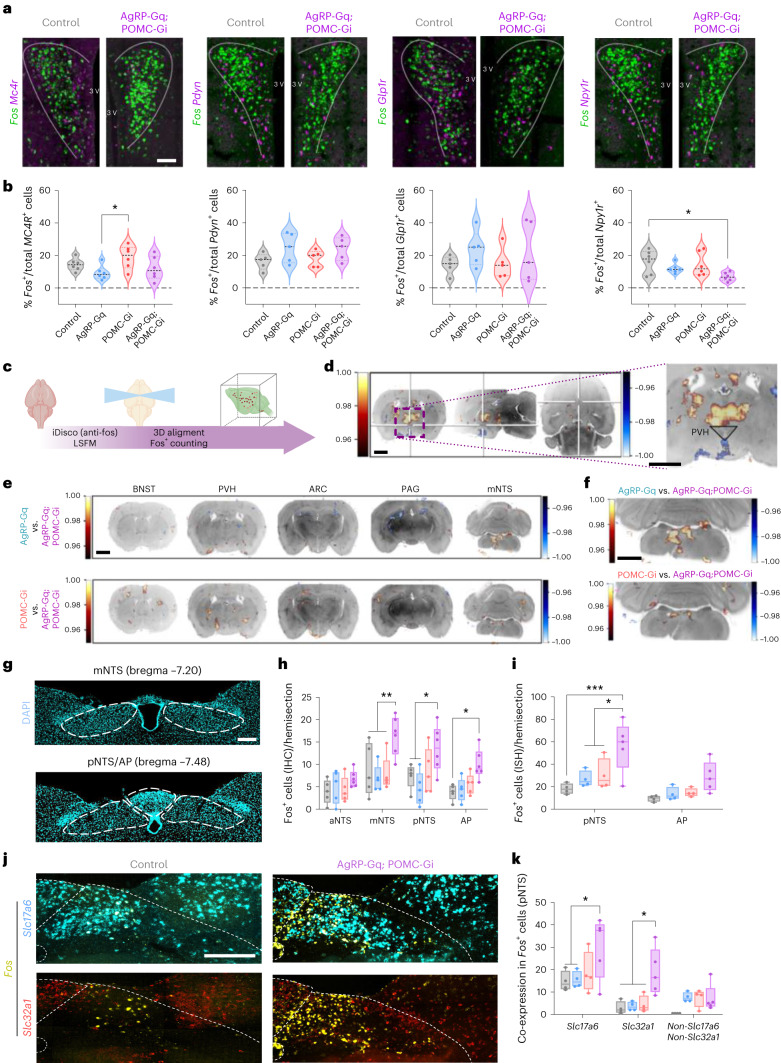
Npy1R^+^ neurons in PVH respond to simultaneous neuronal input from AgRP and POMC neurons. **a**, Representative images (**a**) and quantification of the percentage of *Mc4r*, *Pdyn*, *Glp1r* and *Npy1r* expressing-neurons in PVH area that colocalize with *Fos* mRNA in CNO-treated mice for 1 h (**b**). *P = 0.0442 AgRP-Gq versus POMC-Gi in *MC4R*^+^ analysis, *P = 0.0402 control versus AgRP-Gq;POMC-Gi in *Npy1R*^+^ analysis. **c**, Schematic of experimental protocol for unbiased whole-brain Fos analysis. **d**, Representative images from Fos volumetric comparison (*n* = 4 mice per group; Methods) between CNO-treated control versus AgRP-Gq mice, showing coronal, sagittal and transcranial sections. Insert shows a coronal section at the PVH area, with increased Fos signal in the thalamus (orange) area and decreased Fos signal in the periventricular area (blue). **e**, Representative coronal images of the Fos volumetric comparisons between AgRP-Gq versus AgRP-Gq;POMC-Gi mice (upper) and POMC-Gi versus AgRP-Gq;POMC-Gi group (lower). **f**, Detailed image of Fos volumetric analysis at the coronal DVC area for each previous comparison. **g**, Representative images of medial (mNTS) and posterior NTS/AP (pNTS) areas from the DVC complex. **h**, Quantification of Fos protein expression in the DVC area from CNO-treated mice for 1 h. **P = 0.005 AgRP-Gq;POMC-Gi versus rest of groups in the mNTS area, *P = 0.0385 AgRP-Gq;POMC-Gi versus control and AgRP-Gq group in the pNTS area; and *P = 0.0362 AgRP-Gq;POMC-Gi versus control in the AP area. **i**, Quantification of cells expressing *Fos* mRNA in pNTS and AP areas from CNO-treated mice for 1 h. *P = 0.0184 AgRP-Gq;POMC-Gi versus AgRP-Gq and POMC-Gi groups, ***P = 0.0004 AgRP-Gq;POMC-Gi versus control. **j**, Representative FISH images of the pNTS area showing the colocalization of *Slc17a6* (blue) and *Slc32a1* (red) markers in *Fos*^+^ (yellow) neurons. **k**, Quantification of *Fos*^*+*^ neurons that co-express *Slc17a6*, *Slc32a1* or none of these genes in the pNTS area. *P = 0.0141 AgRP-Gq;POMC-Gi versus control and AgRP-Gq for *Slc17a6*, *P = 0.059 AgRP-Gq;POMC-Gi versus rest of the groups for *Slc32a1*. Data are the average ± s.e.m. of the percentage of positive cells per hemisection of PVH area in **b**; and the mean ± s.e.m. of total positive cells per hemisection of NTS area in **h**, **i** and **k**. Biological replicates are: **b**, *n* = 6 mice per group for *MC4R*^+^ and Npy1R^+^, and 5 for *Pdyn*^+^ and *Glp1R*^+^ analysis; **c**–**e**, *n* = 4 mice per group; **h**, *n* = 5 control, 5 AgRP-Gq, 5 POMC-Gi and 6 AgRP-Gq;POMC-Gi mice; **i** and **k**, *n* = 4 control, 4 AgRP-Gq, 4 POMC-Gi and 5 AgRP-Gq;POMC-Gi mice. Box plots indicate the median ± minimum/maximum values and include data points of individual mice. Statistical significance was determined by one-way ANOVA followed by Tukey’s test for **b**, and two-way ANOVA followed by Sidak’s test for **h**, and Tukey’s test for **i** and **k**. White scale bar in **a**, **g** and **j** , 100 µm. Black scale bar in **d**–**f**, 1,500 µm. Figure 3c created with BioRender.com. IHC, immunohistochemistry; LSFM, light sheet fluorescence microscopy. Source data

**Extended Data Fig. 5:**
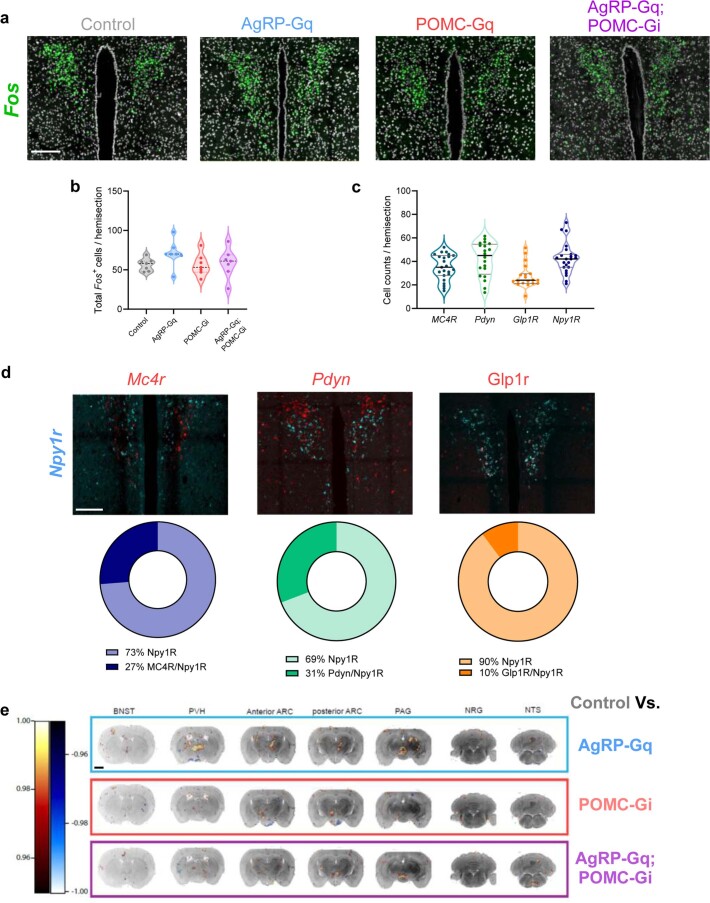
Distribution of PVH neuronal subpopulations and unbiased analysis of activated areas resulting from activation of AgRP- and inhibition of POMC neurons. **a**, Representative images showing the distribution of *Fos*^+^ cells in the PVH after CNO treatment of the four experimental groups. **b**, Quantification of total *Fos*^+^ cells/hemisection in PVH area from CNO-treated mice for 1h. **c**, Quantification of total cells expressing *Mc4r*, *Pdyn, Glp1r* and *Npy1r* genes per PVH/hemisection area. **d**, Anatomical distribution and composition of Npy1R subpopulations that coexpress *M*, *Pdyn*, *Glp1r* markers in the medial PVH area, and quantification of percentage of total *Npy1R*^+^ population that coexpress another marker. **e**, Representative images of coronal sections across the whole brain highlighting areas with higher (yellow-white areas) or lower (blue-white) probability of containing Fos^+^ cells resulting from the statistical comparison of volumetric Fos distribution between control mice versus AgRP-Gq (top row, blue), POMC-Gi (middle row, red) and AgRP-Gq;POMC-Gi mice (lower row, magenta) after CNO treatment for 1h. Data represent average ± s.e.m of total cell counts per hemisection, from *n* = 7 mice/group in **b,c**, and from *n* = 4 mice/group in **d**, **e**. Statistical test: ordinary one-way ANOVA test for **b**. Source data and further details of statistical analyses are provided as a Source Data file. Scale bar = 100 µm. Source data

Next, we aimed to identify which downstream areas and neuronal populations are able to integrate simultaneous functionally opposing neuronal inputs from both AgRP and POMC neurons. We focused our attention on the PVH, an area composed of heterogeneous neurons^25^ that have a key role in the regulation of feeding, metabolic, stress and hormonal responses^26–29^. We quantified the number of activated cells after CNO treatment by assessing *Fos* mRNA expression in the medial PVH. Here, the total number of *Fos*^+^ cells did not change between experimental groups, although there was a trend to increase in the AgRP-Gq group (Extended Data Fig. 5a,b). Next, we analysed the activation state of specific PVH subpopulations that are critical for food intake regulation: MC4R^PVH^, Pdyn^PVH^ and Glp1R^PVH^ neurons^30–32^. We additionally analysed Npy1R^PVH^ neurons, a subpopulation that has been shown to respond to multiple behavioural states^33^ and also mediates AgRP neuron-controlled activation of the hypothalamic–pituitary–adrenal axis and liver autophagy^28^. Chemogenetic activation of AgRP neurons did not significantly affect the activation state of any of the PVH subpopulations analysed (Fig. 3a,b), although we observed a non-significant trend to reduce the activation state of MC4R^PVH^ and Npy1R^PVH^ neurons (Fig. 3b). Similarly, CNO-induced silencing of POMC neurons did not significantly alter the activation state of the PVH populations analysed (Fig. 3b). These results suggest that reducing POMCergic tone does not rapidly modify the activation state of PVH downstream neurons consistent with the temporal scale of POMC neuron-dependent control of food intake (Fig. 2b). However, CNO-treated AgRP-Gq;POMC-Gi mice showed a significant decrease in *Fos*^+^ cell counts only in the Npy1R^PVH^ subpopulation compared to controls (Fig. 3b), suggesting that Npy1R^PVH^ neurons can integrate simultaneous signals originating from both AgRP and POMC neurons. Of note, Npy1R^PVH^ neurons show a small percentage of overlap with the other PVH subpopulations analysed (Extended Data Fig. 5c,d).

Additionally, we investigated whether the interaction between AgRP and POMC neurons cooperatively regulates the activation state of other downstream areas. For this, we used an unbiased whole-brain analysis of activated areas after reciprocal chemogenetic manipulation of AgRP and POMC neurons (Fig. 3c). We combined the whole-brain clearing tissue protocol iDisco^34^ with three-dimensional (3D) fluorescence image capture and use of custom-made registration algorithms to align each brain 3D structure to a unique brain reference, previously registered onto the mouse brain atlas. Then, we developed an algorithm to localize and count Fos^+^ signals within 3D-registered images. Volumetric Fos^+^ signals were used to perform the statistical comparisons between experimental groups to identify brain areas that present a significant change in the number of Fos^+^ cells (Fig. 3d and Extended Data Fig. 5e). We noticed that areas densely innervated with AgRP and POMC projections, such as the PVH or the bed nucleus of the stria terminalis area, did not reveal significant differences between these experimental conditions (Fig. 3d).

To identify regions that respond only to the coordinate actions of activated AgRP and silenced POMC neurons, we compared the volumetric Fos^+^ signal of the AgRP-Gq;POMC-Gi group versus the signals from isolated chemogenetic intervention on AgRP-Gq or POMC-Gi mice (Fig. 3e). This analysis pointed to a specific area in the posterior hindbrain, the dorsal vagal complex (DVC), where AgRP-Gq;POMC-Gi mice presented higher Fos^+^ immunoreactivity than the individual chemogenetic interventions (Fig. 3f).

### Npy1R^PVH^ neurons participate in the regulation of feeding behaviour

To further define the specific nature of neurons under the control of the coordinate action of AgRP and POMC neurons in the DVC area, we specifically analysed Fos protein and *Fos* mRNA expression in coronal sections covering the DVC area (Fig. 3g) in the different groups of experimental animals (Supplementary Fig. 3a,b). Here, AgRP-Gq;POMC-Gi mice presented higher numbers of Fos^+^ cell counts in the medial and posterior nucleus tractus solitarius (NTS) area, as well as in the area postrema (AP), compared to CNO-treated control, AgRP-Gq and POMC-Gi mice (Fig. 3h). Quantification of *Fos*^*+*^ mRNA expression showed similar results for the posterior NTS area, although it did not reach statistical significance in the AP (Fig. 3i and Supplementary Fig. 3b). *Fos* mRNA expression in AgRP-Gq;POMC-Gi mice was also increased compared to isolated chemogenetic intervention on AgRP-Gq and POMC-Gi mice (Fig. 3i). We also characterized the co-expression of glutamatergic (*Slc17a6*) and GABAergic (*Slc32a1*) markers in *Fos*^+^ cells at the posterior NTS/AP area (Fig. 3j). In control, AgRP-Gq and POMC-Gi mice, the *Fos*^+^ cells were mainly glutamatergic (*Slc17a6*^+^). However, the AgRP-Gq;POMC-Gi group presented a significant increase in the number of glutamatergic (*Fos*^+^, *Slc17a6*^+^) and GABAergic (*Fos*^+^, *Slc32a1*^+^) activated neurons (Fig. 3k).

**Fig. 4:**
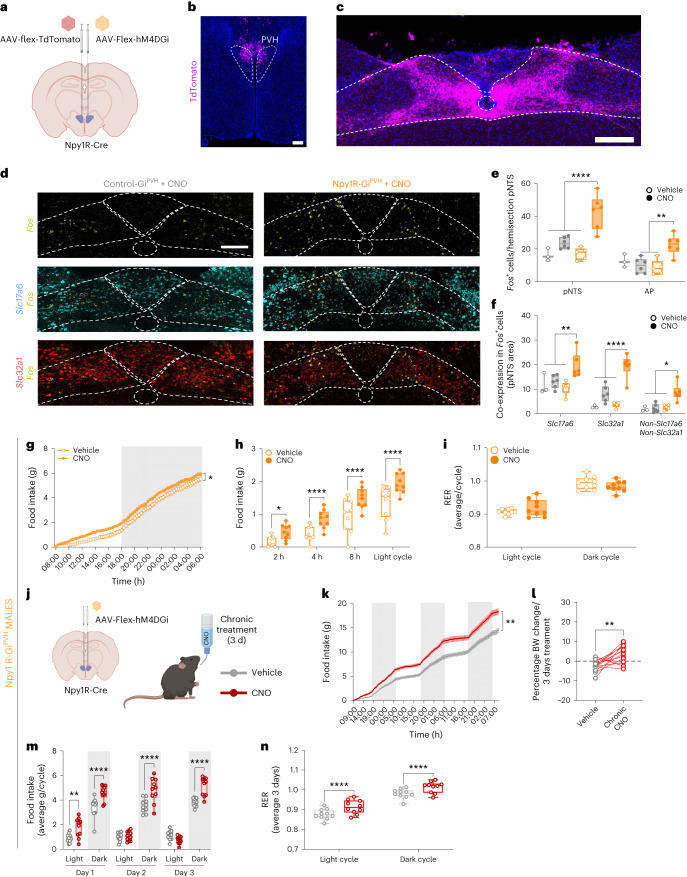
Inhibition of Npy1R^PVH^ neurons activates glutamatergic and GABAergic neurons at the NTS area and promotes food intake in male mice. **a**, Schematic of AAV delivery into the PVH area of Npy1R-Cre^+/−^ mice. **b**, Representative image of AAV-Flex-tdTomato injection site into the PVH area (*n* = 3 independent replicates). **c**, Image of posterior NTS/AP area showing axonal projections from Npy1R^PVH^-tdTomato^+^ neurons (*n* = 2 independent replicates). **d**, Representative FISH images of pNTS area showing the colocalization of *Fos* (yellow) and *Slc17a6* (cyan) or *Slc32a1* (red) markers in control-Gi^PVH^ and Npy1R-Gi^PVH^ mice treated with CNO for 1 h in the absence of food. **e**, Quantification of total *Fos*^+^ cells in the pNTS and AP areas in control-Gi^PVH^ (grey bars, *n* = 3 vehicle-treated and 6 CNO-treated mice) and Npy1R-Gi^PVH^ mice (orange bars, *n* = 5 vehicle-treated and 6 CNO-treated mice) after vehicle (open bars) or CNO injection (solid bars). **P = 0.022 and ****P < 0.001. **f**, Quantification of the total *Fos*^+^ cells that co-expressed *Slc17a6* and *Slc32a1* markers in pNTS area after vehicle or CNO injection. (*n* = 3 vehicle-treated and 6 CNO-treated control-Gi^PVH^ mice; and *n* = 5 vehicle-treated and 6 CNO-treated Npy1R-Gi^PVH^ mice). *P = 0.0164, **P = 0.0052 and ****P < 0.001. **g**, Cumulative food intake during 24 h in ad libitum-fed Npy1R-Gi^PVH^ mice treated with vehicle (open dots) and CNO (solid dots; *n* = 9 male mice), *P = 0.0253. **h**, Quantification of delta food intake (∆ = CNO − vehicle) at different intervals after vehicle or CNO injection in Npy1R-Gi^PVH^ mice (*n* = 9 male mice), *P = 0.013 and ****P < 0.001. **i**, Average of RER in light and dark cycles from Npy1R-Gi^PVH^ mice treated with vehicle or CNO (*n* = 9 male mice). **j**, Scheme of chronic CNO treatments in Npy1R-Gi^PVH^ mice. **k**, Cumulative food intake along 3 d in mice treated chronically with CNO or vehicle in drinking water (*n* = 10 male mice) **P = 0.0026. **l**, Percentage of body weight gain after 3 d chronic treatment with vehicle or CNO (*n* = 15 male mice), **P = 0.0016. **m**, Average of food intake during light and dark cycles along the 3-d chronic treatment with vehicle or CNO in drinking water (*n* = 10 male mice), **P = 0.0051, ****P < 0.001. **n**, Average respiratory coefficient (RER) from 3 d of chronic treatment during light and dark cycles (*n* = 10 male mice), ****P < 0.0001. Data are presented as the average ± s.e.m. for each biological replicate. Box plots indicate the median ± minimum/maximum and include data points of individual mice. Statistical significance was determined by a two-tailed paired *t*-test for **l**; two-way ANOVA followed by Tukey’s test for **e**, **f** and **n**; and Bonferroni’s test for **g**–**m**. Scale bar, 100 µm. Figure 4a,j created with BioRender.com. Data from female mice are displayed in Extended Data Fig. 6. Source data

We then investigated whether the NTS/AP area receives direct neuronal input from Npy1R^PVH^ neurons. We injected a Cre-dependent AAV-Flex-tdTomato into the PVH area of Npy1R-Cre mice to visualize tdTomato-positive axonal projections (Fig. 4a,b). Npy1R^PVH^ neurons send wide axonal projections throughout the brain (Supplementary Fig. 4), reaching the bed nucleus of the stria terminalis area and the medial hypothalamic area, where high numbers of fibres surround the main nuclei (ARC, DMH and VMH) and strongly innervate the median eminence, but also reaching the PVT, TVA, PAG and CA1 regions. Finally, within the hindbrain region, a high number of fibres is present in the posterior NTS area, as recently reported^35^, while the AP and the dorsal motor nucleus of the vagus (DMV) areas exhibit lesser axonal projections (Fig. 4c).

**Extended Data Fig. 6:**
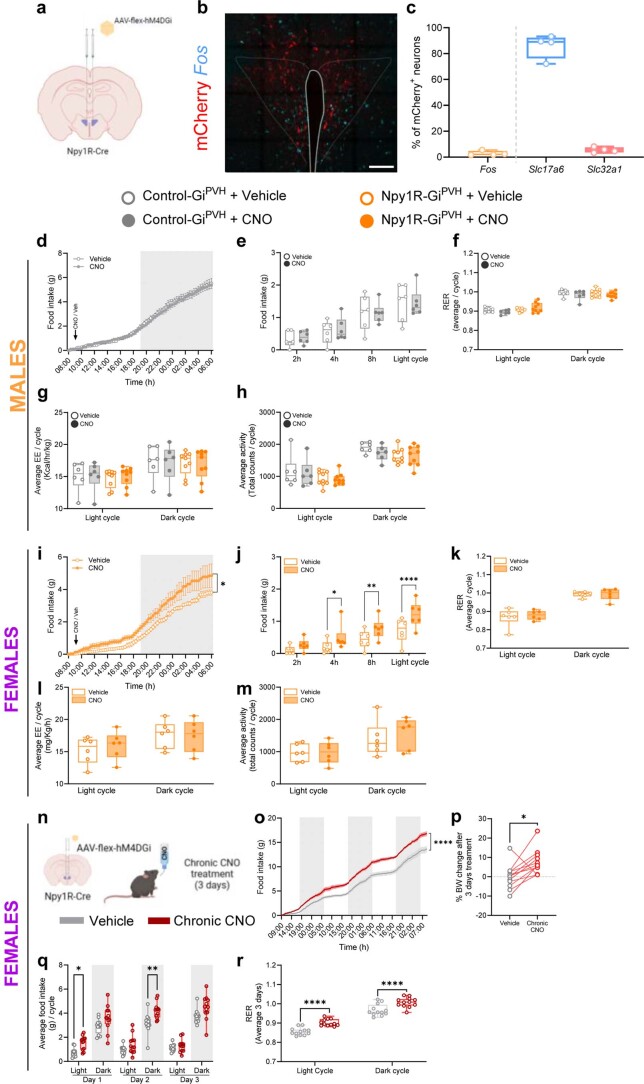
Chemogenetic inhibition of Npy1RP^VH^ neurons does not affect energy expenditure. **a**, Scheme of AAV-flex-hM4DGi injection into PVH area of Npy1R-Cre^+/−^ mice. **b**, Representative image of NPy1R^PVH^ neurons that express hM4DGi-mCherry protein (red) showing almost no colocalization with *Fos* mRNA marker (blue) after CNO injection (3 mg/kg, 1h). **c**, Quantification of chemogenetic-induced silencing of mCherry-expressing Npy1R^+^ neurons in PVH area, and characterization of neurotransmitter nature by colocalization with *Slc17a6*^+^ or *Slc32a1*^*+*^ markers (*n* = 4 male mice). **d**, Cumulative food intake curves after vehicle or CNO injection in Control-Gi^PVH^ (Npy1R-Cre ^−/−^) mice (*n* = 6 male mice) **e**, Food intake during the time intervals from Control-Gi^PVH^ Gi male mice (*n* = 6 male mice) treated with vehicle (open bars) or CNO (closed bars). **f**, Average RER during the light and dark cycles in Control-Gi and Npy1R-Gi mice (*n* = 6 and 9 male mice, respectively) treated with vehicle (open bars) or CNO (closed bars. **g**, Average of energy expenditure (EE) along the light and dark cycles in Control-Gi and Npy1R-Gi mice (*n* = 6 and 9 male mice, respectively) treated with vehicle (open bars) or CNO (closed bars). **h**, Average of activity counts during the light and dark cycles in Control-Gi and Npy1R-Gi mice (*n* = 6 and 9 male mice, respectively) treated with vehicle (open bars) or CNO (closed bars). **i**, Cumulative food intake curves after vehicle or CNO injection from female Npy1R-Gi mice treated with vehicle (open dots) or CNO (closed dots) (*n* = 6 female mice), *P = 0.0364 Veh. Vs. CNO. **j**, Food intake during the time intervals from Npy1R-Gi female mice (*n =* 6 female mice) treated with vehicle (open bars) or CNO (closed bars); *P = 0.102, **P = 0.0034 and ****P < 0.001 Veh vs. CNO. **k**, Average of respiratory coefficients (RER) for light and dark cycle from female Npy1R-Gi mice treated with vehicle (open bars) or CNO (closed bars) (*n* = 6 female mice). **l**, Average of energy expenditure (EE) for light and dark cycle from female Npy1R-Gi mice treated with vehicle (open bars) or CNO (closed bars) (*n* = 6 female mice). **m**, Average of activity counts during light and dark cycle from female Npy1R-Gi mice treated with vehicle (open bars) or CNO (closed bars). **n**, Scheme of the experimental design. **o**, Cumulative food intake along 3 d of chronic treatment with vehicle (grey lines) or CNO (red lines) in drinking water for Npy1R-Gi female mice (*n* = 12 mice) ****P < 0.0001. **p**, Percentage of body weight gain after 3 d with chronic treatment with vehicle or CNO in female Npy1R-Gi mice (*n* = 12 female mice) *P = 0.0102. **q**, Average of food intake during light and dark cycles along the 3-day chronic treatment with vehicle or CNO in drinking water (*n* = 12 female Npy1R-Gq mice) *P = 0.0251 and **P = 0.0019. **r**, Average respiratory coefficient (RER) from 3 d of chronic treatment during light and dark cycles in female Npy1R-Gi mice (*n* = 12 female mice). ****P < 0.0001. Data represent average ± s.e.m of the biological replicates. Boxplots indicate median ±min/max and include data points of individual mice entering the analysis. Statistical test: two tailed paired T-test for **p**, 2-way ANOVA for **d – r**; followed by Bonferroni’s test for **I**, **k**, **o** and **r**; and Sidak’s test for **j**, **l**, **m** and **q**. Source data and further details of statistical analyses are provided as a Source Data file. White scale bar = 100 µm in **a**, **d**. Black scale bar = 1500 µm in **e**. Created with BioRender.com. Source data

We interrogated whether the reduction of neuronal activity of Npy1R^PVH^ neurons results in changes in the activity of posterior NTS/AP neurons by injecting AAV-Flex-hM4DGi-mCherry into the PVH area of Npy1R-Cre^+/−^ mice (Npy1R-Gi^PVH^ mice) or their Npy1R-Cre-negative littermates (Control-Gi^PVH^; Fig. 4a). CNO-treated Npy1R-Gi^PVH^ mice showed almost complete silencing of Npy1R^PVH^-mCherry^+^ neurons (Extended Data Fig. 6b,c). Exploration of the posterior NTS area revealed that CNO-induced silencing of Npy1R^PVH^ neurons resulted in an increased number of *Fos*^+^ glutamatergic (*Fos*^+^, *Slc17a6*^+^) and GABAergic (*Fos*^+^, *Slc32a1*^+^) neurons (Fig. 4d–f).

Next, we investigated whether the inhibition of Npy1R^PVH^ neurons affects the regulation of food intake. We treated Npy1R-Gi^PVH^ male and female mice with CNO at the beginning of the light cycle, and we found a significant increase in food intake that started faster in male (2 h) than in female (4 h) mice and continued along the light cycle (Fig. 4g,h and Extended Data Fig. 6i,j). CNO-injected control-Gi^PVH^ male mice showed no significant change in food intake and other metabolic parameters (Extended Data Fig. 6d–h). The increase in food intake in Npy1R-Gi^PVH^ mice during the light cycle was accompanied by a non-significant increase in RER during the same period (Fig. 4i and Extended Data Fig. 6k), without further alterations in EE or total activity measurements (Extended Data Fig. 6g,h,l,m). These results confirm that Npy1R^PVH^ neurons participate in the regulation of feeding behaviour, probably by acting as a relay station between AgRP and POMC neurocircuits and other further downstream areas, potentially including the posterior NTS/AP area.

Additionally, we investigated whether the inhibition of Npy1R^PVH^ neurons may modify feeding behaviour in the long term by exploring the effect of chronic silencing of Npy1R^PVH^ neurons (Fig. 4j). Therefore, we added fresh CNO to water bottles each day to sustain a chronic CNO treatment, similarly to previous reports^36,37^. Chronic Npy1R^PVH^ inhibition increased food intake across the 3 d of CNO compared to vehicle treatment (Fig. 4k and Extended Data Fig. 6o), resulting in increased body weight at the end of the CNO treatment (Fig. 4l and Extended Data Fig. 6p). Although increased food consumption was seen in mice during the light cycle after the first exposure to CNO, the main effect of increased food intake was observed during the dark cycles, especially in male mice (Fig. 4m and Extended Data Fig. 6q). In line with the increased feeding, RER increased in both the light and dark phase (Fig. 4n and Extended Data Fig. 6r).

### Identification of NTS neurons activated by reciprocal AgRP/POMC regulation

**Fig. 5:**
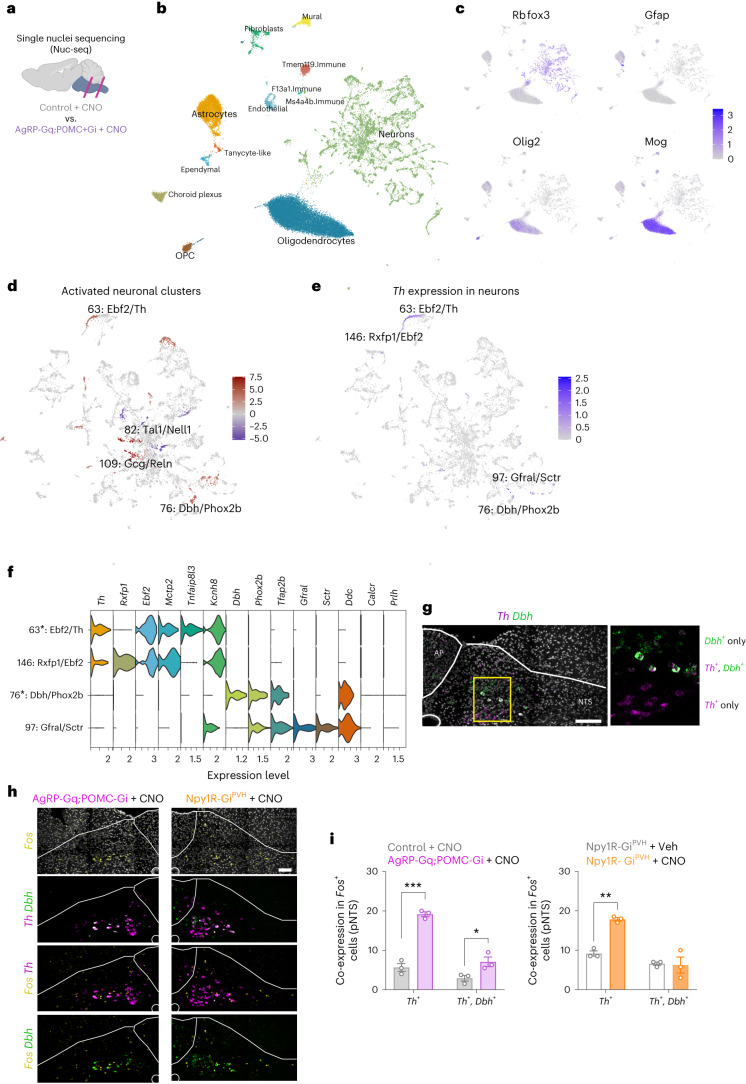
*Th*^+^ neurons are activated in the posterior NTS area by the interplay of AgRP and POMC neurocircuits. **a**, Schematic of the single-nucleus sequencing experiment from hindbrain samples. **b**, UMAP visualization of cell-type composition of hindbrain single-nucleus sequencing data (*n* = 2 control and 2 AgRP-Gq;POMC-Gi mice). **c**, UMAP showing the distribution of main cell-type markers. Colour corresponds to log-normalized expression **d**, UMAP highlighting the top activated (red) or inhibited (blue) neuronal clusters. Colour displays a score based on differential gene expression between AgRP-Gq:POMC-Gi and control mice of ten core IEGs (Methods). **e**, UMAP showing the expression of the *Th* gene in neuronal clusters. **f**, Violin plot of expression level of main marker genes associated with the selected *Th*^+^ and *Dbh*^+^ clusters. The asterisk denotes clusters with higher-ranking IEGs. **g**, Representative image showing the spatial distribution of neurons that express *Th*^+^, *Dbh*^+^ and *Th*^+^*Dbh*^+^ by mRNA FISH in the posterior NTS area (*n* = 3 mice per group). **h**, Representative images showing the distribution and colocalization of *Fos*^+^ (yellow) neurons that express *Th*^+^ (magenta) and *Dbh*^+^ (green) from AgRP-Gq;POMC-Gi and Npy1R-Gi^PVH^ mice treated with CNO for 1 h. **i**, Quantification of *Fos*^+^ colocalization with total *Th*^+^ and *Th*^+^*Dbh*^+^ neurons in posterior NTS from AgRP-Gq;POMC-Gi and Npy1R-Gi^PVH^ mice relative to their respective control (*n* = 3 mice per group), *P = 0.0448, **P = 0.0011 and ***P = 0.0004. Data represent the mean ± s.e.m. for each group and treatment. Statistical significance was determined by two-way ANOVA followed by Bonferroni’s multiple-comparison test. Scale bar, 100 µm. Figure 5a created with BioRender.com. Source data

**Extended Data Fig. 7:**
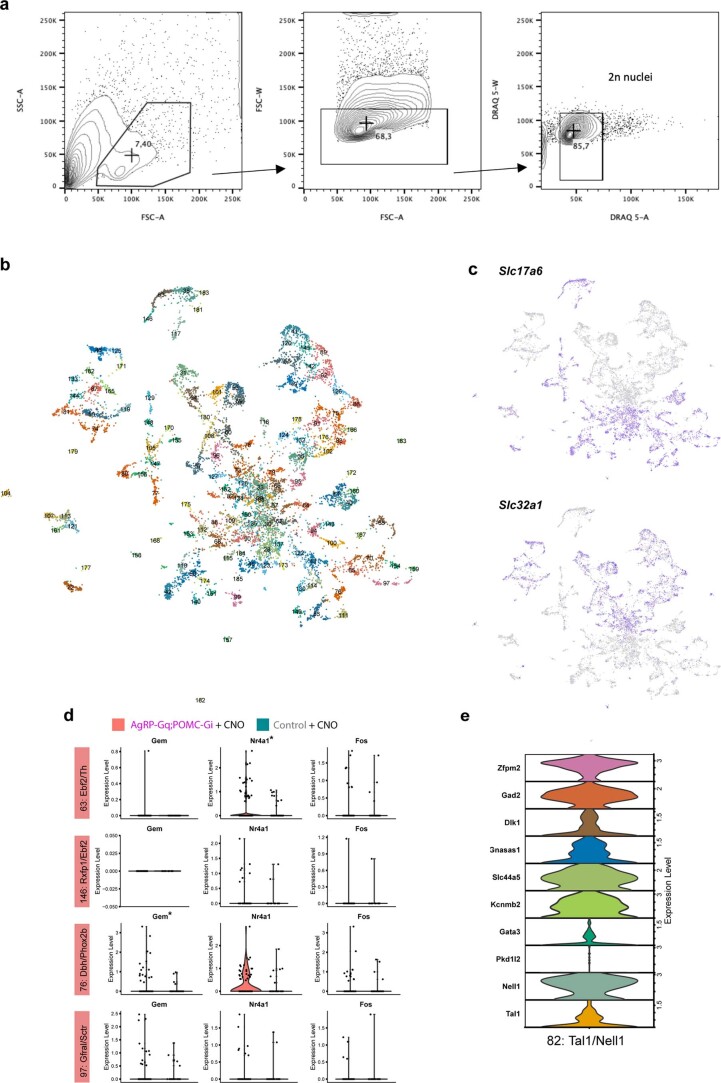
Identification of activated neuronal cells in the NTS/AP area using sc-seq data. **a**, Examplary gating strategy for sorting by flow cytometry: Nuclei determined by side scatter area (SSC-A) vs. forward scatter area (FSC-A). Singlets determined by FSC-A vs. forward scatter width (FSC-W). 2n nuclei determined by DRAQ5 fluorescence (DRAQ5 width vs. DRAQ5 area). **b**, UMAP showing the distribution of the 136 neuronal cluster, of a total of 186 clusters across all cell type. c, UMAP showing the distribution of glutamatergic and GABAergic neuronal clusters based on *Slc17a6* and *Slc32a1* gene expression, respectively. **d**, Violin-plots showing the expression of IEGs of the four neuronal cluster selected based on their high score in the activated IEGS analysis. (Gem*) denotes significant difference of expression between AgRP-Gq;POMC-Gi + CNO (red violins) vs. Control + CNO (green violins) comparison. e, Expression of main marker genes in the individual nuclei from Cluster 82. Source data and further details of statistical analyses are provided as a Source Data file. Source data

To further define the molecular nature of *Fos*^+^ cells in the posterior NTS/AP area that respond to simultaneous chemogenetic modulation of AgRP and POMC neurons (Fig. 3f,k), we isolated the posterior part of the hindbrain region that contains the DVC complex from male and female CNO-treated AgRP-Gq;POMC-Gi and control mice and performed single-nucleus RNA sequencing^38^ (Fig. 5a and Extended Data Fig. 7a). Analysis of the data revealed a clear separation based on specific cell identities on a uniform manifold approximation and projection (UMAP) plot (Fig. 5b, see Data Availability and Code Availability in Methods). Annotation of cell types showed a majority of clusters from neuronal origin followed by oligodendrocytes and astrocytes (Fig. 5b), exemplified by the enriched expression of main cell markers: *Rbfox3* for neurons, *Gfap* for astrocytes, *Olig2* for oligodendrocytes and oligodendrocyte precursors cells and *Mog* for mature oligodendrocytes (Fig. 5c). In the following, we focused on neuronal clusters (136 of 186 total clusters; Extended Data Fig. 7b), which exhibited clear segregation between glutamatergic (*Slc17a6*^*+*^) and GABAergic (*Slc32a1*^*+*^) neurons (Extended Data Fig. 7c). We used a binomial mixed model to determine differentially expressed genes between CNO-treated AgRP-Gq;POMC-Gi and control mice for each cluster^39^ and focused on a set of ten immediate early genes (IEGs; Methods) to rank potentially activated candidate clusters. We selected the four neuronal clusters with the highest IEG rank in AgRP-Gq;POMC-Gi compared to control samples for further analysis (Fig. 5d and Extended Data Fig. 7d). Three of them were glutamatergic, whereas cluster 82:Tal1/Nell1 expressed GABAergic markers. This latter cluster may represent the GABAergic neurons that respond and become activated in our chemogenetic interventions and might participate in the regulation of food intake (Figs. 3k and 4f). However, the molecular characterization of cluster 82:Tal1/Nell1 did not reveal any single gene marker to further characterize these neurons by FISH experiments (Extended Data Fig. 7e).

Of the glutamatergic candidates, cluster 109:Gcg/Reln had the highest IEG score. However, chemogenetic activation of Gcg^NTS^ neurons has been shown to reduce food intake^40^. Therefore, we focused our attention on clusters 63:Ebf2/Th and 76:Dbh/Phox2b, which may coincide with neuronal populations that have been proposed to regulate feeding behaviour in various physiological states, such as hunger^41^ or glucodeprivation^42^. Analysis of tyrosine hydroxylase (*Th)* gene expression in our neuronal UMAP revealed four related *Th*^*+*^ clusters (Fig. 5e): 63:Ebf2/Th and 76:Dbh/Phox2b, which exhibited activation based on the IEG rank, and clusters 97:Gfral/Sctr and 146:Rxtp2/Ebf2, which showed no activation based on the IEG rank (Extended Data Fig. 7d). Detailed analysis of the gene expression profiles of these four *Th*^*+*^ clusters revealed differences in the expression of *Th* and *Dbh* genes (Fig. 5f): the clusters 63:Ebf2/Th and 146:Rxtp2/Ebf2 exhibited high *Th* expression but almost no *Dbh* expression, while the cluster 76:Dbh/Phox2b showed high *Dbh* and *Ddc* expression, suggesting a catecholaminergic nature, although this cluster showed little *Th* expression. Finally, cluster 97:Gfral/Sctr, presented both modest expression of *Th* and *Dbh* genes and a strong *Ddc* expression, but exhibited a low IEG rank. Furthermore, the anorexigenic markers *Calcr* and *Prlh* were absent in the *Th*+ clusters 63:Ebf2/Th and 146:Rxtp2/Ebf2, although both markers exhibited low expression in clusters 76:Dbh/Phox2b and 97:Gfral/Sctr (Fig. 5f).

These results suggest that *Th*^+^*Dbh*^*−*^ neurons could be a population distinct from *Th*^+^*Dbh*^+^ neurons, and that both may have different roles in the regulation of food intake. Therefore, we assessed the distribution of *Th*^+^ and *Th*^+^*Dbh*^+^ neurons in the posterior NTS/AP area by mRNA FISH (Fig. 5g). Based on this analysis, we localized a small spatial cluster of Th-only neurons (*Th*^+^*Dbh*^−^*)* in the ventral area of the pNTS. This area is surrounded by a dorsal subset of neurons that exhibited co-expression of *Th*^+^*Dbh*^+^ genes. In the more dorsal area of the pNTS, we identified a sparse cluster of Dbh-only neurons (*Dbh*^+^*Th*^−^; Fig. 5g). Apart from those spatial clusters, we observed a sparse distribution of *Th*^+^, *Dbh*^+^ and *Th*^+^*Dbh*^+^ neurons in the medioventral area of the pNTS.

Finally, we interrogated whether *Th*^+^ and *Th*^+^*Dbh*^+^ neurons are activated by assessing *Fos* expression by mRNA FISH in samples from AgRP-Gq;POMC-Gi and Npy1R-Gi^PVH^ mice treated with CNO compared to their respective controls (Fig. 5h). The quantification of activated, *Fos*^+^*Th*^+^ neurons in the NTS area showed a significant increase in CNO-treated AgRP-Gq;POMC-Gi compared to control mice (Fig. 5i). Nevertheless, the level of activation of catecholaminergic *Th*^+^*Dbh*^+^ neurons also showed a significant increase (Fig. 5h,i). In parallel, chemogenetic inhibition of Npy1R^PVH^ neurons resulted in a significant increase of *Fos*^+^*Th*^+^ cell counts in the pNTS area compared to vehicle-treated Npy1R-Gi mice, without an increase in *Fos* mRNA expression in *Th*^+^*Dbh*^+^ neurons (Fig. 5i). Collectively, these results suggest that Th^NTS^ neurons may receive the neuronal inputs initiated by the simultaneous and coordinated interplay between AgRP and POMC neurocircuits.

### Systemic insulin sensitivity is differentially regulated by the opposing interaction between AgRP and POMC neurocircuits

**Fig. 6:**
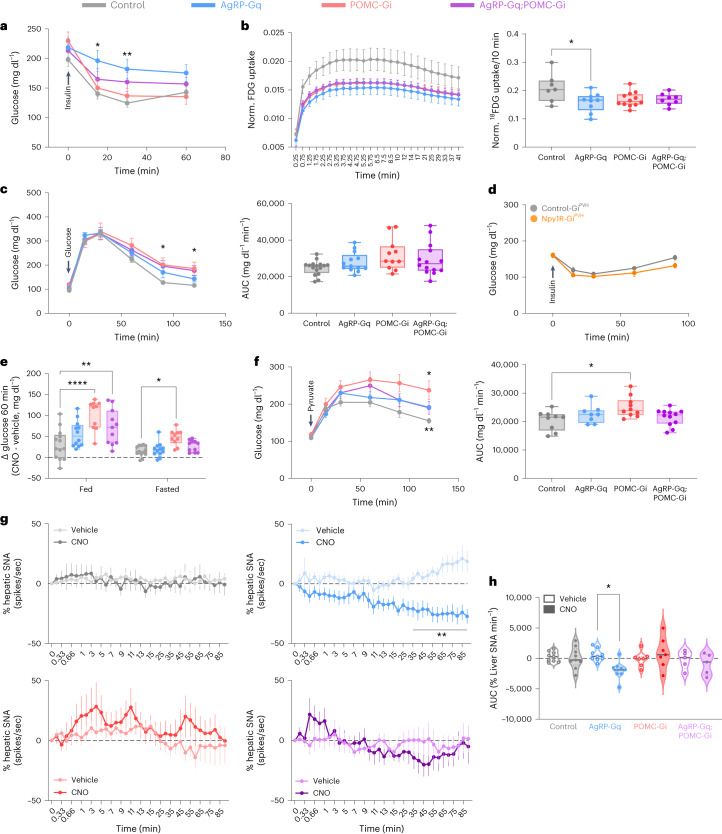
Insulin sensitivity and liver SNA are controlled by the antagonistic interplay between the AgRP and POMC neurocircuits in male mice. **a**, Insulin tolerance test performed in ad libitum-fed male mice treated with CNO for 1 h before insulin injections. *P = 0.0125 at 15 min and **P = 0.0094 at 30 min in AgRP-Gq versus control mice. **b**, Curves of ^18^FDG uptake in BAT tissue (right) from male mice treated with CNO for 1 h and AUC quantification of ^18^FDG uptake (left) during 10 min in BAT tissue from male mice. *P = 0.0234 AgRP-Gq versus control. **c**, Glucose tolerance test and quantification of AUC in 16-h-fasted male mice treated with CNO for 1 h before glucose injection. *P = 0.0309 at 90 min and **P = 0.0487 at 120 min in POMC-Gi versus control. **d**, Insulin tolerance test performed in ad libitum-fed male control-Gi^PVH^ (grey lines) and Npy1R-Gi^PVH^ (orange lines) male mice. **e**, Changes in glucose levels during 1 h after CNO injection in ad libitum-fed and 16-h fasted mice, *P = 0.0377 POMC-Gi versus control in fasting, **P = 0.0028 AgRP-Gq;POMC-Gi versus control and ****P < 0.0001 POMC-Gi versus control. **f**, Glucose levels and quantification of AUC during a pyruvate tolerance test performed in 16-h-fasted male mice after 1 h CNO injection. *P = 0.0418 POMC-Gi versus control, **P = 0.0021 AgRP-Gq versus control, and *P = 0.0199 POMC-Gi versus control in AUC. **g**, Changes in hepatic SNA after intravenous (i.v.) administration of vehicle or CNO (1 mg per kg body weight) in anaesthetized mice. Data are presented as the mean with 95% confidence interval. **h**, Quantification of the AUC of changes in liver SNA. *P = 0.0273 vehicle versus CNO in AgRP-Gq group. Data are presented as the average ± s.e.m. for each mouse, except on **g**. Box plots indicate the median ± minimum/maximum and include data points of individual mice. Biological replicates are: **a**, *n* = 20 control, 12 AgRP-Gq, 15 POMC-Gi and 13 AgRP-Gq;POMC-Gi male mice; **b**, *n* = 7 control, 9 AgRP-Gq, 12 POMC-Gi and 8 AgRP-Gq;POMC-Gi male mice; **c**, *n* = 16 control, 13 AgRP-Gq, 11 POMC-Gi and 14 AgRP-Gq;POMC-Gi male mice; **d**, *n* = 5 control-Gi and 9 Npy1R-Gi male mice; **e**, *n* = 13 control, 12 AgRP-Gq, 10 POMC-Gi and 11 AgRP-Gq;POMC-Gi male mice; **f**, *n* = 9 control, 8 AgRP-Gq, 10 POMC-Gi and 12 AgRP-Gq;POMC-Gi male mice; **g** and **h**: *n* = 9 control, 9 AgRP-Gq, 7 POMC-Gi and 5 AgRP-Gq;POMC-Gi mice. Statistical significance was determined by one-way ANOVA followed by Tukey’s multiple-comparison test for **b**, **c** and **h**; two-way ANOVA followed by Tukey’s multiple-comparison test for **a**, **e** and **f**, and by Bonferroni’s multiple-comparison test for **c**, **d** and **g**. Data from female mice are displayed in Extended Data Fig. 8. Source data

**Extended Data Fig. 8:**
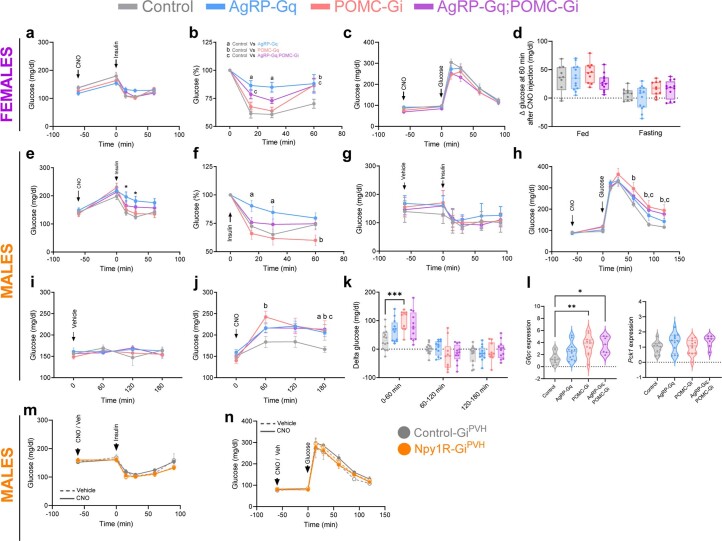
Female mice data and extended data for glucose homeostasis assessment. **a**, Insulin tolerance test (ITT) performed in *ad-libitum* fed CNO-treated female mice for 1h before insulin injections. **b**, Glucose levels during ITT experiment relative to basal glucose levels prior to insulin injection. Letters indicate statistical significance between experimental groups: (a) AgRP-Gq vs. control, **** P < 0.001 15 min, ***P = 0.0001 30 min; (b) POMC-Gi vs. control, *P = 0.0131 60 min; and (c) AgRP-Gq;POMC-Gi vs. control, *P = 0.0133 30 min, *P = 0.0224 60 min. **c**, Glucose tolerance test (GTT) in 16h fasted CNO-treated female mice for 1h before glucose injection. **d**, Delta glucose levels in 60 min after CNO injection in female mice in *ad libitum* fed or 16h fast condition. **e**, Complete curves of glucose levels during ITT in *ad libitum* fed male mice. *P = 0.006815min and *P = 0.004930 min, AgRP-Gq vs. control. **f**, Glucose levels during ITT experiment relative to basal glucose levels prior to insulin injection. (a) AgRP-Gq vs. control, **P = 0.0076 15 min and **P = 0.0030 30 min; (b) POMC-Gi vs. control, *P = 0.041 60 min. **g**, Complete glucose levels curves during ITT protocol in *ad libitum* fed male mice treated with vehicle prior to insulin injection. **h**, Complete curves of glucose levels during an GTT protocol in 16h fasted CNO-treated for 1h male mice. (b) POMC-Gq vs. control, *P = 0.013 60 min, **P = 0.0034 90 min and ** P = 0.006 120 min; (c) AgRP-Gq;POMC-Gi vs. control, *P = 0.0107 90 min and * P = 0.0263 at 120 min. **i**, Glucose excursion induced by vehicle injection in *ad libitum* fed male mice **j**, Glucose excursion induced by CNO injection in *ad libitum* fed male mice. (a) AgRP-Gq vs. control, *P = 0.34 180 min; (b) POMC-Gi vs. control, **P = 0.0013 60 min, *P = 0.022 180 min; and (c) AgRP-Gq;POMC-Gi vs. control, **P = 0.0083 180 min. **k**, Changes in glucose levels in 60 min intervals after CNO injection in *ad libitum* fed male mice. ***P = 0.0008 POMC-Gi vs. control. **l**, Expression of gluconeogenic enzymes *G6P* and *Pck1* from liver extracts from CNO-treated male mice for 1h. **P = 0.0055 POMC-Gi vs. control, *P = 0.0139 AgRP-Gq;POMC-Gi vs. control. **m**, Glucose levels during ITT in *ad libitum* fed Control-Gi^PVH^ (grey lines) and Npy1R-Gi^PVH^ (orange lines) male mice treated with vehicle (dashed lines) or CNO (solid lines) 1h prior to insulin injection. **n**, Glucose excursion during GTT protocol in 16h fasted male Control-Gi and Npy1R-Gi mice treated with vehicle (dashed lines) or CNO (solid lines). Data are indicated as mean ± s.e.m. from the following biological replicates for Control, AgRP-Gq, POMC-Gi and AgRP-Gq;POMC-Gi groups, respectively: **a**, **b** (*n* = 17, 13, 15, 13 female mice), **c** (n = 16, 15, 14, 12 female mice), **d** (*n =* 10, 13, 10, 12 female mice), **e**, **f** (*n =* 20, 12, 15, 13 male mice), **g** (*n =* 5, 4, 7, 9 male mice), **h** (*n =* 16, 13, 10, 14 male mice), **i** (n = 8, 7, 8, 8 male mice), **j** (*n =* 14, 12, 10, 12 male mice), **k** (n = 14, 12, 10, 12 male mice), **l** (*n =* 10, 12, 11, 7 male mice), **m** and **n** (*n = 5* control-Gi and 9 Npy1R-Gi male mice). Boxplots indicate median ± min/max and include data points of individual mice entering the analysis. Statistical test: 2-way ANOVA followed by Tukey’s multiple **a – n**, except ordinary one-way ANOVA for **l**. Letters indicate statistical significance between: (a) Control vs. AgRP-Gq; (b) Control vs. POMC-Gi; (c) Control vs. AgRP-Gq;POMC-Gi. Source data and further details of statistical analyses are provided as a Source Data file. Source data

In addition to control of feeding, AgRP neurocircuits play a role in the regulation of insulin sensitivity, which is critical to maintaining precise control of glucose homeostasis^43^. Acute optogenetic or chemogenetic activation of AgRP neurons resulted in systemic insulin resistance, partially caused by reduced glucose uptake in BAT^18,22^. Therefore, we explored whether the interplay with POMC neurocircuits modulates the effect of activated AgRP neurons on insulin sensitivity. To this end, we performed insulin tolerance tests in mice treated with CNO for 1h without access to food. In male and female mice, CNO-induced AgRP neuron activation decreased overall insulin sensitivity compared to CNO-treated control mice (Fig. 6a and Extended Data Fig. 8a,b,e,f), as previously reported^22^. Silencing of POMC neurons does not result in altered insulin sensitivity compared to control mice (Extended Data Fig. 8b,f). Notably, we observed an intermediate phenotype in male and female AgRP-Gq;POMC-Gi mice, where simultaneous inhibition of POMC neurons partially rescues the insulin resistance phenotype resulting from activated AgRP neurons (Fig. 6a and Extended Data Fig. 8b,f).

To further investigate the mechanism underlying this phenomenon, we quantified the rate of radioactively labelled glucose (^18^F-fluorodeoxyglucose (^18^FDG)) uptake in BAT depots in anaesthetized male mice placed into a micro-positron emission tomography (PET)/computed tomography (CT) scanner 1 h after CNO injection. ^18^FDG uptake in BAT was reduced in all experimental groups compared to control mice, although only the AgRP-Gq mice presented a significant decrease compared to control mice, which was no longer significant in AgRP-Gq;POMC-Gi mice (Fig. 6b), consistent with the effects observed on systemic insulin sensitivity.

Next, we investigated whether glucose homeostasis is also controlled by the reciprocal interaction between AgRP and POMC neuronal activity. We performed a glucose tolerance test in 16-h-fasted mice after injecting CNO 1 h before the glucose administration. Glucose excursions and area under the curve (AUC) calculation (Fig. 6c and Extended Data Fig. 8c,h) showed no differences between males and females from any experimental group, except at 90–120 min, where male POMC-Gi mice exhibited significantly increased glucose concentrations compared to the control group (Fig. 6c).

Based on our previous results, we interrogated whether Npy1R^PVH^ neurocircuits also participate in the regulation of glucose homeostasis. We assessed insulin and glucose tolerance tests in Npy1R-Gi^PVH^ male mice 1 h after the CNO treatment and found no differences compared to control littermates (control-Gi^PVH^) or vehicle-treated mice (Fig. 6d and Extended Data Fig. 8m,n). These results indicate that Npy1R^PVH^ neurons integrate the effects of AgRP and POMC neurons to modulate feeding behaviour but not glucose homeostasis.

However, we observed a significant increase in glucose levels following CNO stimulation in POMC-Gi male mice, but not in the AgRP-Gq, AgRP-Gq;POMC-Gi or Npy1R-Gi^PVH^ groups or in female mice (Fig. 6e and Extended Data Fig. 8d,m,n). This effect is observed in both fed and overnight fasted mice, but not when mice were injected with vehicle (Extended Data Fig. 8i). Then, we examined temporal changes in glucose levels after CNO injection in fed male mice in the absence of subsequent food supply. Results confirmed that only POMC-Gi male mice showed a significant increase in glucose levels during the first 60 min compared to control group, followed by a steady decrease over the next 120 min (Extended Data Fig. 8j,k). These data indicate that isolated chemogenetic inhibition of POMC neurons promotes a short-term increase in glucose levels.

Therefore, we examined liver gluconeogenesis as a possible source of acute blood glucose elevation. We performed a pyruvate tolerance test in male mice fasted for 16h after stimulation with CNO, and we observed a significantly sustained glucose production in POMC-Gi mice compared to the control group (Fig. 6f). Previous reports showed that chronic (6h), but not acute (30 min), AAV-directed chemogenetic inhibition of POMC neurons resulted in increased gluconeogenic capacity^44^. However, we achieved a similar result in our transgenic model as early as 1h after inhibition of POMC neurons. Additionally, we analysed the gene expression of gluconeogenic key enzymes G6Pase (*G6PC1)* and PEPCK (*Pck1*) in the liver of 1h CNO-treated male mice (Extended Data Fig. 8l). G6Pase expression was elevated in POMC-Gi and AgRP-Gq;POMC-Gi groups, although PEPCK expression did not change between mice of the different experimental groups. These results indicate that isolated silencing of POMC neurons may predominantly promote liver gluconeogenesis resulting in acute hyperglycaemia, although this effect is not present when AgRP neurons are simultaneously activated. Collectively, our data point to an antagonistic effect of reciprocally regulated AgRP and POMC neurocircuits over BAT and liver responses.

### Liver SNA is modulated by the interplay between AgRP and POMC neurons

The ability of AgRP and POMC neurons to regulate metabolism in peripheral organs relies at least in part on their rapid modulation of the autonomous nervous system^13,17,18,45^. Based on our results and published observations, we hypothesized that the interaction between AgRP and POMC neurocircuits differently modulates the net efferent sympathetic nervous system (SNS) tone in comparison to the isolated effects derived from each neurocircuit alone. To investigate this, we measured the liver SNA in anaesthetized mice followed by treatment with vehicle and CNO (Fig. 6g,h). The analysis of SNA spikes in the control group showed no differences between treatments, indicating that CNO does not affect liver SNA tone in control mice. CNO-induced chemogenetic activation of AgRP neurons resulted in a significant reduction in SNA activity compared to vehicle-treated mice, which aligns with previous results in BAT^18^ (Fig. 6g,h). CNO-induced inhibition of POMC neurocircuits does not produce significant changes in liver SNA activity. Finally, simultaneous chemogenetic intervention on AgRP and POMC neurons showed that silenced POMC neurons prevent the reduction of liver SNS activity driven by AgRP neuron activation (Fig. 6g,h), similarly to the control of systemic insulin sensitivity (Fig. 6a and Extended Data Fig. 8b,f). These results support the notion that the antagonistic interaction between the neuronal activity of both ARC neurocircuits ultimately may control hepatic responses during the feeding–fasting transition by the precise regulation of SNS tone subserving the liver.

### HFD-induced obesity disrupts the food intake modulation exerted by AgRP and POMC neuron interactions

**Fig. 7:**
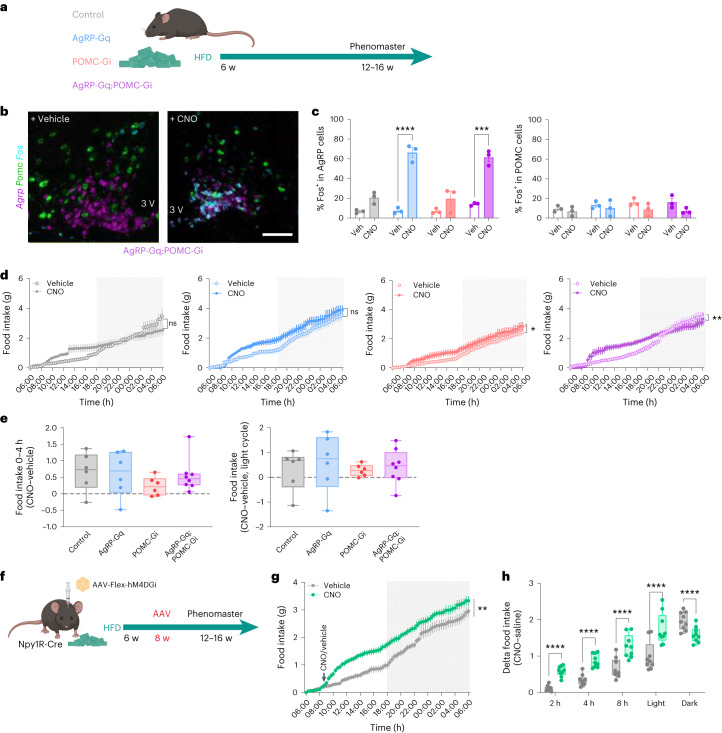
Obesity disrupts the coordinated regulation from AgRP and POMC neurocircuits over food intake and insulin sensitivity in male mice. **a**, Scheme for HFD-induced obesity. **b**, Representative images of the ARC area from AgRP-Gq;POMC-Gi HFD-fed mice treated with vehicle or CNO showing the colocalization of *Fos* mRNA (cyan) with *Agrp* (magenta) and *Pomc* (green) neurons. **c**, Percentage of *Fos* colocalization on total *AgRP* (left) and *POMC* (right) neurons ***P = 0.0001 AgRP-Gq;POMC-Gi vehicle versus CNO; and ****P < 0.0001 AgRP-Gq vehicle versus CNO data represent the average ± s.e.m. of cell counts per hemisection from *n* = 3 mice for each group and treatment. **d**, Cumulative food intake curves from HFD-fed male mice treated with vehicle or CNO at the beginning of the light phase *P = 0.0362 for POMC-Gi, vehicle versus CNO, and **P = 0.0095 AgRP-Gq;POMC-Gi, vehicle versus CNO. **e**, Delta food intake (Δ = CNO – vehicle) for the 0–4-h interval and during the light cycle from HFD-fed male mice. **f**, Schematic of experimental design in Npy1R-Gi^PVH^ mice. **g**, Cumulative food intake curves from HFD-fed male Npy1R-Gi^PVH^ mice treated with vehicle or CNO, **P = 0.0024 vehicle versus CNO. **h**, Food intake at time intervals in HFD-fed Npy1R-Gi male mice, ****P < 0.0001 vehicle versus CNO. Data are represented as the average ± s.e.m. per mouse and treatment from the following biological replicates**: d** and **e,** *n* = 6 control, 6 AgRP-Gq, 6 POMC-Gi and 8 AgRP-Gq;POMC-Gi HFD-fed male mice; **g** and **h**, *n* = 9 HFD-fed Npy1R-Gi male mice. Box plots indicate the median ± minimum/maximum and include data points of individual mice. Statistical significance was determined by one-way ANOVA followed by Tukey’s test for **e**, two-way ANOVA followed by Sidak’s multiple-comparison test for **c**, **d** and **h** and Bonferroni’s test for **g**. Scale bar, 100 µm. Figure 7a,f created with BioRender.com. Data from female mice are displayed in Extended Data Fig. 9c,d. Source data

**Extended Data Fig. 9:**
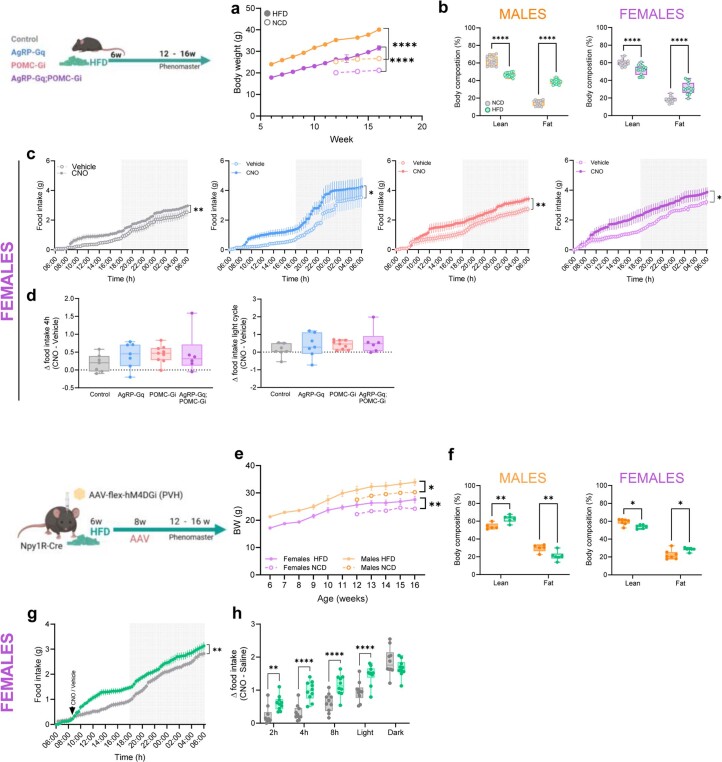
Female and extended data for impact of HFD-induced obesity on food intake modulated by reciprocal interplay between AgRP and POMC neurons. **a**, Body weight curves during HFD exposition in males and females, and comparison with normal body weight curves in normal chow diet (NCD) fed mice. ****P < 0.0001. Mice from the four experimental groups were pooled together. (*n* = 8 mice/group, diet and sex, 2 mice per each experimental group). **b**, Body composition changes in 16weeks old male and female mice fed with NCD or HFD. Mice from the four experimental groups were pooled in balanced ratios (*n* = 16 male and 14 female mice/diet). **c**, Cumulative food intake curves from HFD-fed female mice treated with vehicle or CNO at the beginning of the light phase. Vehicle vs. CNO: **P = 0.0094 control, *P = 0.0278 AgRP-Gq, **P = 0.0061 POMC-Gi, *P = 0.0278 AgRP-Gq;POMC-Gi. (*n* = 7 control, 7 AgRP-Gq, 9 POMC-Gi, 6 AgRP-Gq;POMC-Gi female mice) **d**, Delta food intake (Δ = CNO – vehicle) for the 0–4h interval and the light cycle interval in HFD-fed female mice. (*n* = 7 control, 7 AgRP-Gq, 9 POMC-Gi, 6 AgRP-Gq;POMC-Gi female mice). **e**, Body weight curves during HFD exposition in male and female Npy1R-Gi mice, and comparison with normal body weight curves in NCD fed mice. *P = 0.0384 male mice, and **P = 0.0089 female mice (*n* = 6 mice/sex and diet). **f**, Body composition changes in 16 weeks old male and female Npy1R-Gi mice treated with NCD or HFD. Males: **P = 0.0085 lean mass, and P = 0.0069 fat mas. Females: *P = 0.0285 lean and *P = 0.013 fat mass. (*n* = 5 NCD- and 6 HFD-fed male mice, 7 NCD- and 6 HFD-fed female mice) **g**, Cumulative food intake curves from HFD-fed Npy1R-Gi^PVH^ female mice and treated with vehicle (grey lines) or CNO (green curves) at the beginning of the light cycle ** P = 0.093. (*n* = 10 female mice). **h**, Food intake at time intervals in HFD-fed Npy1R-Gi^PVH^ female mice. **P = 0.0012 at 2h, and ****P < 0.0001 at 4h, 8h and light cycle interval. Data represents mean ± s.e.m for each experimental group mice. Boxplots indicate median ±min/max and include data points of individual mice entering the analysis Statistical test: ordinary one-way ANOVA in **d**; 2-way ANOVA Mixed-effects model in **a**, **e**; 2-way ANOVA followed by Tukey′s multiple comparisons test in **b;** and Bonferroni’s test in **c**, **g**, and Sidak’s test in **f** and **h**. Source data and further details of statistical analyses are provided as a Source Data file. Created with BioRender.com. Source data

Lastly, we interrogated whether obesity affects the effects of the coordinated reciprocal interplay between AgRP and POMC neurocircuits in the regulation of feeding and metabolism. We exposed mice from the four experimental groups to 60% high-fat-diet (HFD) for 6–12 weeks (Fig. 7a), which resulted in increased body weight and adiposity (Extended Data Fig. 9a,b). We first assessed whether CNO-induced activation of DREADDS was still functional in HFD-fed mice after acute CNO injection (Fig. 7b). CNO injections induced a similar percentage of activated AgRP neurons in AgRP-Gq and AgRP-Gq;POMC-Gi groups (Fig. 7c). However, CNO treatment did not achieve a significant reduction in POMC activation state indicating that HFD feeding had already inhibited these neurons (Fig. 7c). Assessment of cumulative food intake over 24 h showed an increase in food consumption, which was significant in POMC-Gi and AgRP-Gq;POMC-Gi male mice (Fig. 7d) and in all female experimental groups (Extended Data Fig. 9c) treated with CNO compared with vehicle, including control mice. However, food intake intervals at 4 h after CNO injection, and along the light cycle, showed no differences between the different experimental groups (Fig. 7e and Extended Data Fig. 9d). These results indicate that HFD-induced obesity may impair the ability of AgRP and POMC neurocircuits to cooperatively increase and prolong food intake, indicating that HFD feeding may already alter their basal activity as previously shown^46–48^. Nevertheless, the CNO effects observed in HFD-fed control mice limit the interpretation of these results.

Lastly, we investigated whether obesity also affected the ability of Npy1R^PVH^ neuron inhibition to increase feeding. To this end, we exposed Npy1R-Cre mice to HFD feeding, which resulted in increased body weight and fat mass accruement (Fig. 7f and Extended Data Fig. 9e,f). Inhibition of Npy1R^PVH^ neurons with one acute CNO injection was enough to rapidly increase food consumption during the light cycle in male and female Npy1R-Gi^PVH^ mice (Fig. 7g,h and Extended Data Fig. 9g,h). The increment in food consumption presents a similar temporal pattern compared with Npy1R-Gi^PVH^ mice in control diet (Fig. 4h and Extended Data Fig. 6j). Altogether, these data suggest that PVH neurocircuits are less impacted by HFD-induced obesity in contrast with the effect of HFD on the coordinated interaction between AgRP and POMC neurocircuits.

## Discussion

AgRP and POMC hypothalamic neurocircuits play an prominent role in the regulation of metabolism as they respond in opposite directions to changes in circulating hormones and nutrients, external cues and synaptic input^16,49^. Bidirectional changes in their neuronal activity are integrated by downstream neuronal populations that, on the one hand, regulate food intake and, on the other hand, physiological adaptations of metabolic organs^50^. Here, we investigated the precise regulation of several metabolic functions governed by the reciprocal interplay between AgRP and POMC neurocircuits and delineated a downstream neurocircuit where the opposing regulation of both cell types cooperates to regulate feeding behaviour.

We established and validated a generally applicable transgenic approach to simultaneously and opposingly regulate two genetically defined neuronal subtypes. Previously, our laboratory had successfully combined non-interacting Cre and Dre recombinases to delineate and characterize specific POMC subpopulations^51^. Here, we targeted each recombinase to functionally opposing neuronal populations (that is, AgRP and POMC neurons) to restrict the expression of opposite chemogenetic receptors in these neurons. Histological examination and electrophysiological recordings confirmed the restricted expression of each chemogenetic receptor and their cell-autonomous response to CNO application, resulting in hyperpolarization of hM4DGi-expressing POMC neurons and depolarization of hM3DGq-expressing AgRP neurons. Interestingly, chemogenetic activation of AgRP neurons did not cause a significant reduction in *Fos*^+^*POMC*^+^ cells in AgRP-Gq mice as revealed by FISH experiments (Fig. 1e). Additional electrophysiological recordings in the absence of synaptic blockers showed that chemogenetic activation of AgRP neurons causes the indirect inhibition of 70% of POMC neurons, with a modest reduction of the POMC membrane potential (Fig. 1i). These results contrast with previous electrophysiological studies using viral-based optogenetic activation of AgRP neurons that resulted in almost complete POMC inhibition^5,10^. Those differences may result from the different cellular mechanisms of optogenetic versus chemogenetic manipulation, as well as the relative expression of each receptor (ChR2 or DREADDs) achieved depending on the method of gene delivery (AAV-mediated versus transgenic). Here, our chemogenetic intervention achieves a moderate activation of 45% of AgRP neurons, similar to the effect of acute ghrelin injection (Extended Data Fig. 4a) and to the percentage of activated AgRP neurons after a short-term fasting period^28^. In fact, previous studies have characterized other GABAergic, non-AgRP neurons in the ARC that also participate in the GABAergic inhibition of POMC neurons^52–54^. Thus, our simultaneous chemogenetic intervention over both ARC populations resulted in a further hyperpolarization of POMC neurons (Fig. 1j) that may recreate the neuronal dynamics in AgRP and POMC neurons during the feeding–fasting transitions, mimicking the effect of additional inhibitory inputs to POMC neurons besides those arising from AgRP neurons.

Functionally, our experiments revealed that isolated chemogenetic activation of AgRP neurons or single chemogenetic inhibition of POMC neurons promotes food intake, but at different timescales. Chemogenetic activation of AgRP neurons in male mice induced a rapid increase in food intake during the first 2 h, while chemogenetic inhibition of POMC neurons increases food intake after 4 h of CNO application. Previous reports showed that acute chemogenetic inhibition of POMC neurons did not modulate food intake after 1 h of CNO administration. In comparison, chronic chemogenetic inhibition for 24 h did effectively increase food intake^5,55^. These differences in POMC temporal inhibition patterns can be attributed to previously non-assayed intermediate time points or to the strategies selected to express inhibitory DREADDs in POMC neurons: AAV-delivered versus our case—transgenic mouse models.

Notably, the simultaneous opposite chemogenetic manipulation on AgRP and POMC neurons showed a more sustained stimulation of feeding compared to the effects of individual chemogenetic manipulation of AgRP or POMC neurons, especially in male mice (Fig. 2a,b). In an attempt to define the molecular underpinnings of this interaction, we investigated neurocircuits where reciprocal AgRP and POMC neuronal inputs converge and cooperate in the control of downstream targets. Given the pivotal role of PVH neurocircuits, especially in the regulation of feeding behaviours, we investigated whether specific PVH neuronal population(s) may respond to simultaneous changes in AgRP and POMC neuronal activity. We found that Npy1R^PVH^ neurons reduced their neuronal activity after simultaneous chemogenetic intervention on AgRP and POMC neurons (Fig. 3b). Additionally, an elegant study revealed that Npy1R^PVH^ neurons integrate the neuronal responses resulting from multiple behavioural states^33^. More recently, feeding regulation has been shown to depend on the precise regulation of cAMP levels in MC4R^PVH^ neurons by the interplay of AgRP and POMC neuronal signalling, where MC4R^PVH^ neurons integrate NPY and α-MSH signals from AgRP and POMC axonal terminals^56^. This biphasic signalling model can similarly act on 30% of Npy1R^PVH^ neurons that co-express MC4R, and contribute to the cooperative regulation of feeding by AgRP and POMC neurocircuits. Nevertheless, it is possible that other mechanism(s) related to decreased glutamatergic transmission by silenced POMC neurons play a role in the modulation of Npy1R^PVH^ neuronal activity. Notably, chemogenetic inhibition of Npy1R^PVH^ neurons during the light cycle results in a significant increase in food intake (Fig. 4g,h), supporting their role as mediators of AgRP and POMC neurons in the control of feeding. This may also be relevant for leptin action, which simultaneously inhibits AgRP neurons and activates POMC neurons.

Npy1R^PVH^ neurons have previously been shown to project axonal terminals to hindbrain areas, including the NTS and the dorsal raphe areas, which control SNA in BAT^57^ (Fig. 4c and Supplementary Fig. 4). Furthermore, our unbiased whole-brain analysis of activated areas revealed an NTS area that rapidly responds to the reciprocal chemogenetic intervention on both ARC populations (Fig. 3e,f). Aligning with these observations, we found that both of our chemogenetic interventions over the interplay of AgRP and POMC neurocircuits, and the inhibition of Npy1R^PVH^ neurons coincided in increasing neuronal activation in the medial and posterior areas of the NTS (Figs. 3i,k and 4e,f). Regarding the identity of those activated NTS neurons, our analysis of single-nucleus sequencing of the hindbrain pointed to two activated neuronal clusters (Fig. 5d) characterized by *Th* and *Dbh* gene expression. Abundant literature has shown that several NTS neuronal subpopulations participate in the aversive suppression of feeding, such as GLP-1, CCK and 5TH2C populations^58^, although recent reports informed about Th^NTS^ and Dbh^NTS^ neuronal populations that actually promoted feeding behaviour in different physiological conditions^41,42^. In both reports, chemogenetic activation of *Th*^+^ or *Dbh*^+^ neurons in the NTS area promotes food intake in the light cycle. Remarkably, Th^NTS^ neurons project axonal terminals towards the mediobasal hypothalamus, reaching AgRP and POMC neurons, and promoting increased food intake through AgRP activation and POMC inhibition via noradrenaline release^42^. Assessment of colocalization between *Fos*, *Th* and *Dbh* mRNA expression in CNO-treated AgRP-Gq;POMC-Gi mice revealed an increase in the activation state of both *Th*^+^ and *Th*^+^*Dbh*^+^ neurons in the posterior NTS. Additionally, the chemogenetic silencing of Npy1R^PVH^ neurons produced a concomitant increase in activated *Th*^+^ neurons, but not in *Th*^+^*Dbh*^+^ neurons (Fig. 5h,i). Thus, we propose that Th^NTS^ neurons may participate in the control of feeding behaviour triggered by the simultaneous activation of AgRP and inhibition of POMC neurons. Th^NTS^ neurons may also act as a second relay station, downstream of Npy1R^PVH^ neurocircuits, that facilitates the cooperative effect of the AgRP and POMC interplay to further promote food intake. However, we cannot exclude that increased feeding upon Npy1R^PVH^ neuron inhibition is mediated via additional or alterative projections of these cells. In addition, our single-nucleus sequencing data following concomitant regulation of AgRP and POMC neurons revealed the activation of *Gcg*^+^ neurons as well as some *Th*^+^*Dbh*^+^ neurons that express the satiety markers PRLH and CalcR. These NTS neurons have been described to project axonal terminals towards the ARC area, affecting AgRP-induced feeding regulation^59^. Nevertheless, these data suggest that concomitant activation of AgRP neurons and inhibition of POMC neurons is also activating anorexigenic circuits in the NTS, possibly through a complex feedback loop that is not directly driving the observed increase in food consumption.

In addition to regulating feeding, activation of AgRP neurons controls other metabolic adaptations, such as overall nutrient use. Activated AgRP neurons modulate the nutrient flux during the early phase of fasting (4 h), promoting an acute increased use of carbohydrates and enhancing foraging behaviour^15^ (Fig. 2h). Interestingly, none of these physiological adaptations during early fasting changed when POMC neurons were inhibited in isolation, pointing towards the dominant role of AgRP neurons in the regulation of metabolic substrate use during the early feeding–fasting transition.

Nutrient flux requires precise coordination to sustain the production of glucose as the main energy source for the CNS during starvation. Here, AgRP neurons have been shown to directly regulate peripheral insulin sensitivity. Ablation of insulin receptors from AgRP neurons reduces insulin’s ability to suppress hepatic glucose production (HGP)^14^, and isolated chemogenetic activation of AgRP neurons resulted in a decrease in systemic insulin sensitivity, as previously reported^18,22^. This effect has been attributed partially to reduced sympathetic outflow to BAT as well as increased production of branched-chain amino acids in the liver^60^. Notably, the concurrent inhibition of POMC neurons with AgRP neuronal activation partially rescued the insulin resistance induced by AgRP neuron activation. Interestingly, this attenuation of AgRP neuron-induced insulin resistance following concomitant POMC neuron inhibition occurred independent of a rescue in BAT glucose uptake, arguing that this partial restoration occurs via alternative mechanisms. Notably, the inhibition of Npy1R^PVH^ neurons does not result in any alteration in insulin or glucose sensitivity (Extended Data Fig. 8c,h), suggesting that Npy1R^PVH^ neurons only participate in feeding regulation.

During the fasting transition, the liver plays an important role in maintaining HGP^61^. AgRP and POMC neurons participate in the control of HGP: previous reports have shown that insulin-mediated inhibition of AgRP neurons is required to reduce HGP^14^, and that optogenetic stimulation of direct POMC axonal projections into the DMV area also modulates HGP^62^. We observed that acute reduction of POMC neuronal activity alone produces a significant rapid increase in systemic glucose levels (Fig. 6e) and promotes higher liver gluconeogenetic capacity (Fig. 6f). These effects could result from the rapid reduction in glutamatergic and α-MSH release in POMC axonal terminals that directly connect with autonomic preganglionic neurons within the DMV and the intermediolateral cell column of the spinal cord (IML)^63^. However, these effects are restrained when AgRP neurons are activated and POMC neurons are simultaneously inhibited, indicating again that the interaction of the two neurocircuits drives the regulation of the liver in a different manner compared to how they individually regulate these responses.

Interestingly, this effect was parallelled by a similar regulation of liver SNA, that is, AgRP neuron activation reduced liver SNA, while POMC neuron inhibition had no effect, yet abrogated the AgRP activation-dependent suppression (Fig. 6g,h). These findings are remarkable because chemogenetic activation of POMC neurons acutely increases liver SNA^17^, but it does not modify renal SNA^50^. Therefore, modulation of insulin sensitivity through the opposing actions of AgRP activation and POMC inhibition may converge in the liver to counteract the development of systemic insulin resistance. Possibly, AgRP activation-dependent regulation of branched-chain amino acid metabolism may provide such a potential divergence point, although further functional studies are required to identify the responsible mechanism(s).

The development of obesity results in the deregulation of several metabolic processes that contribute and reinforce the increase in adiposity levels^2^. HFD-induced obesity also affects the metabolic signalling-dependent interplay created by the opposite neuronal regulation of AgRP and POMC neurocircuits (Fig. 7d,e). Interestingly, the ability of Npy1R^PVH^ neurons to modulate food intake was not affected in HFD-fed mice (Fig. 7h and Extended Data Fig. 9h), which suggests that PVH neurocircuits are suitable candidates to identify new druggable targets that will allow for a more effective modulation of feeding behaviours in individuals with obesity.

In conclusion, our data reveal different types of interactions between reciprocal AgRP and POMC neurocircuit activity: (i) cooperative regulation of food intake across different timescales, (ii) predominant short-term control of nutrient utilization via AgRP neuron activation, and (iii) partially opposing roles in the control of insulin sensitivity, gluconeogenesis and hepatic SNA regulation. The Npy1R^PVH ^→ Th^NTS^ neurocircuit provides a plausible substrate for the cooperative regulation of downstream neurons via simultaneous reciprocal regulation of AgRP and POMC neurons that may ultimately regulate food consumption. Nevertheless, the neurocircuitry basis for the observed antagonistic regulations of other metabolic responses will have to be defined in future experiments.

## Methods

### Mice

#### Animal care

All animal procedures were conducted according to the protocols approved by the local government authorities (Bezirksregierung Köln). Permission to maintain and breed mice as well as for all the experimental protocols used in this study was issued by the Department for Environment and Consumer Protection – Veterinary Section, Cologne, North Rhine-Westphalia, Germany. Mice were group housed (3–5 animals per cage) in a controlled environment regarding humidity and temperature (22–24 °C) on a 12-h light–12-h dark cycle. Mice had ad libitum access to water and to a standard rodent chow diet (ssniff, V1154-704), containing 57% of calories from carbohydrates, 34% calories from protein and 9% calories from fat. Additionally, mice were fed ad libitum with a HFD (ssniff, E15742-350) containing 21% calories from carbohydrates, 19% calories from protein and 60% calories from fat.

All experiments were performed in adult mice, aged 12–20 weeks, and both sexes were assayed. Mice were randomly distributed before receiving experimental treatments, always considering balanced sex and body weight distribution in all experimental groups. Chemogenetic experiments were designed in cross-experimental design, ensuring that each mouse received both the chemogenetic actuator and the vehicle. Mice that presented a deviation more than twice the s.e.m. from the average body weight of their sex and experimental group were removed from the experiments. Data from male mice are shown in Figs. 1–7, while data obtained from female mice are displayed in the Extended Data figures. Brain sections were obtained from male and female mice. Liver tissue extracts were taken from male mice.

#### Transgenic mice

All mouse lines were established on a C57BL/6 background. Mouse lines were described previously: AgRP-IRES-Cre^64^, Rosa26-CAG-lox-STOP-lox-hM3DGq-GFP^18^, POMC-Dre^21^ and Npy1R-Cre^65^. Additionally, the Rosa26-CAG-rox-STOP-rox-hM4DGi-ZsGreen line was generated in a similar way as described before^21^.

#### Generation of experimental mice

Heterozygous male AgRP-IRES-Cre^+/−^ mice were bred with homozygous female R26-floxSTOP-flox-hM3DGq^fl/fl^ mice, and heterozygous female POMC-Dre^+/−^ mice were bred with homozygous male R26-rox-STOP-rox-hM4DGi^fl/fl^ mice for three generations. Then, male AgRP-IRES-Cre^+/−^; R26-flox-STOP-flox-hM3DGq^fl/fl^ were bred with female POMC-Dre^+/−^; R26-rox-STOP-rox-hM4DGi^fl/fl^ mice. All offspring mice were heterozygous for hM3DGq^fl/−^ and hM4DGi^fl/−^, with the following four experimental groups, based on the distribution of Cre^+^ and Dre^+^ alleles: control group (AgRP-IRES-Cre^−/−^;hM3DGq^fl/−^;POMC-Dre^−/−^;hM4DGi^fl/−^), AgRP-Gq group (AgRP-IRES-Cre^+/−^;hM3DGq^fl/−^;POMC-Dre^−/−^;hM4DGi^fl/−^), POMC-Gi group (AgRP-IRES-Cre^−/−^;hM3DGq^fl/−^;POMC-Dre^+/−^;hM4DGi^fl/−^) and AgRP-Gq;POMC-Gi group (AgRP-IRES-Cre^+/−^;hM3DGq^fl/−^;POMC-Dre^+/−^;hM4DGi^fl/−^). For the specific experiment in Fig. 1i, a special breeding strategy was used to avoid the hM4DGi^fl/fl^ allele to allow for chemogenetic activation of AgRP-Cre neurons and the possibility to recombine fluorescent protein in POMC-Dre neurons by AAV injection: AgRP-Gq* group (AgRP-IRES-Cre^+/−^;hM3DGq^fl/−^;POMC-Dre^+/−^).

#### Chemogenetics

Mice were injected with CNO (3 mg per kg body weight; Abcam, ab141704) or vehicle (0.9% NaCl, 3% DMSO) intraperitoneally (i.p.) at the start of each experiment. Mice were handled and injected i.p. with 100–200 µl 0.9% NaCl for 3–4 d before the experiment for habituation.

#### Chronic chemogenetic treatment

CNO was diluted into drinking water bottles, which were change every morning. The average drinking volume in our experimental mice was 3.7 ml per day (average 28g) for male mice and 3.5 ml per day (average 25g) for female mice. No differences were observed between experimental mouse lines. To achieve a continuous CNO concentration of 3 mg per kg body weight, a working solution of 5 mg ml^−1^ DMSO was prepared and 45 µl was added to 10 ml of fresh drinking water (DMSO final concentration was <1%), creating a final CNO concentration of 22.5 µg ml^−1^.

### Physiological measurements

#### Metabolic chambers

Food intake, EE and activity were measured using an open circuit indirect calorimetry system (PhenoMaster TSE Systems). Mice were previously single housed and acclimatized in training cages for 3 d before data acquisition to adapt them to the systems’ food and water dispensers. On the experimental day, 2.5–3 h after the beginning of the light cycle, mice were given an i.p. injection of CNO or vehicle and immediately returned to their cages. For measurements in the absence of food, mice were provided with a clean cage at the beginning of the light phase on the experimental day. Three hours later, food was removed, and mice were injected i.p. with CNO. Fat and lean mass were determined using an IVIS SpectrumCT scanner (Caliper LifeScience) as previously described^21^.

#### Insulin tolerance test

Random-fed male mice from 12–16 weeks of age were used in a crossover design, alternating CNO or vehicle injection after 1 week, and experiments were performed as previously described^22^. Mice received a clean cage on the day before the experiments, and the food holder and food spills were carefully removed on the experimental day at the beginning of the light cycle. Basal blood glucose levels were measured (Contour Next, Bayer HealthCare). Mice were injected with CNO (3 mg per kg body weight) or vehicle, and 1 h later, mice were injected with insulin (0.75 U per kg body weight). Blood glucose was measured from tail blood samples at 0, 15, 30, 60 and 90 or 120 min after insulin injection.

#### Glucose tolerance test

Male mice aged between 14 and 16 weeks were fasted for 16 h and used in a crossover design, alternating CNO or vehicle injection after 1 week. Basal blood glucose was measured at the beginning of the light cycle. Mice were injected with vehicle (3% DMSO in saline serum) or CNO (3 mg per kg body weight, i.p.), and 1 h later, glucose (2 g per kg body weight, i.p.) was injected. Blood glucose levels was measured at 0, 15, 30, 60, 90 and 120 min after glucose injection.

#### Pyruvate tolerance test

Male mice aged between 16 and 18 weeks were fasted for 16 h and used in a crossover design, alternating CNO or vehicle injection after 1 week. Basal blood glucose levels were measured at the beginning of the light cycle. Mice were injected with vehicle or CNO (3 mg per kg body weight, i.p.) and 1 h later, pyruvate (2 g per kg body weight, i.p.) was injected. Blood glucose was measured at 0, 15, 30, 60, 90 and 120 min after pyruvate injection.

### Tissue analysis

#### Tissue collection

Approximately 3 h into the light phase, male mice were given an i.p. injection of CNO and food was removed. After 60 min, mice were euthanized, and brains were quickly removed and sectioned using a brain block. Subsequently, BAT and liver depots were collected and snap frozen in liquid nitrogen.

#### Fluorescence in situ hybridization

Mice were deeply anaesthetized and transcardially perfused with 0.9% saline, followed by ice-cold 4% phosphate-buffered paraformaldehyde. Brains stored in 20% sucrose were cut at 14–20 µm on a cryostat (Leica), and sections encompassing whole hindbrain area and hypothalamus were mounted on SuperFrost Plus Gold slides (Thermo Fisher) and stored at −80 °C. RNAscope technology (Advanced Cell Diagnostics) was used for the simultaneous detection of combinations of mRNA markers, following the protocol detailed in ref. ^22^. RNAscope probes are listed in the Supplementary Fig 5.

#### Brain immunostaining

Frozen slides were processed as shown in ref. ^22^. Primary and secondary antibodies used are listed in the Supplementary Fig. 5.

#### Imaging and quantification

Images were captured using a confocal Leica TCS SP-8-X microscope, equipped with a ×40/1.30 oil objective, using tile scans and *z*-stacks (optical section of 1.0 μm). Laser intensities for the different channels were kept constant throughout the imaging process in each experiment at a resolution of 1,024 × 1,024 and a scan speed of 400 ms. Maximum intensity projections were made in Fiji (National Institutes of Health), and the DAPI signal was adjusted with respect to contrast and brightness. Images were edited in Fiji with regards to brightness and contrast. The settings were applied equally across all images. Colocalization of mRNA markers were counted manually with Fiji Cell counter plugin using DAPI signal for validation of colocalization of mRNA markers, and 3–6 hemisections per animal were counted blind to the genotype and the treatment of the animals. For AgRP and POMC axonal projections and Fos protein immunostaining images, a Zeiss Imager M2 fluorescence microscope was used with ×10 and ×4 objectives using the Tile Scan function.

#### qPCR

Total RNA from frozen liver tissues was isolated, transcribed and analysed following the protocol detailed in ref. ^22^. Data were analysed using the ΔCt method, using hypoxanthine guanine phosphoribosyl transferase (*Hprt*, Mm01545399_m1) coupled with VIC fluorescent reporter as the housekeeping gene to normalize within each sample. The TaqMan probes used were glucose-6-phosphatase (*G6pc*, Mm00839363_m1) and phosphoenolpiruvatekinase (*Pck1*, Mm01247058_m1) coupled with a FAM fluorescent reporter.

### Electrophysiological experiments

#### Viral injections during stereotaxic surgeries

For ARC injections, male and female mice from the four experimental groups received a bilateral injection of 250 nl of a 1:0.7 mixture of AAV1-CAG-Frex-ZsGreen and AAV1-flex-TdTomato (Addgene, 28306) into the ARC area at 8–9 weeks of age. Virus expression was allowed for 3–4 weeks before the mice were euthanized for electrophysiological recordings. Bregma coordinates used for ARC injections were: AP, −1.500 mm; ML, ±0.200 mm; DV, −5.80 mm. For PVH injections, male and female Npy1R-Cre mice received a bilateral injection of 50–75 nl of AAV8-hSyn-DIO-hM4DGi-mCherry (Addgene, 44362) at 8–9 weeks of age. Virus expression was allowed for 4 weeks before experiments were performed. Bregma coordinates used for PVH were: AP, −0.7 mm; ML, ±0.150 mm; DV, −4.70 mm. Mice with no clear mCherry signal at the PVH area were excluded for further analysis.

#### Animals and brain slice preparation

The electrophysiological experiments were carried out essentially as described^18,52^. Perforated patch-clamp recordings were performed from genetically marked AgRP and POMC neurons in coronal slices (270 µm) containing the ARC from adult POMC-Gi or AgRP-Gq;POMC-Gi male and female mice. Neurons were identified by their anatomical location in the ARC and by their ZsGreen or tdTomato fluorescence. Unless otherwise stated, the artificial cerebrospinal fluid contained 10^−4^ M picrotoxin (P1675, Sigma-Aldrich), 5 × 10^−6^ M CGP (CGP-54626 hydrochloride, BN0597, Biotrend), 5 × 10^−5^ M DL-AP5 (dl-2-amino-5-phosphonopentanoic acid; BN0086, Biotrend) and 10^−5^ M CNQX (6-cyano-7-nitroquinoxaline-2,3-dione; C127, Sigma-Aldrich) to block GABAergic and glutamatergic synaptic input. Perforated patch-clamp experiments were conducted using protocols modified from previous studies. The used DMSO concentration (0.1–0.3%) had no noticeable effect on the investigated neurons. CNO was bath-applied at a flow rate of ~2.5 ml min^−1^ at a concentration of 3 µM for 5 min.

#### Data analysis

Data analysis was performed with Spike2 (CED), Igor Pro 6 (Wavemetrics) and Prism (version 5.0c; GraphPad Software). To classify a neuron as responsive to CNO application, changes in the membrane potential (mV) induced by CNO were analysed. The mean and standard deviation of the membrane potential was calculated for 2-min intervals before and at the end (minutes 4 and 5) of the CNO application and compared by two-tailed paired *t*-tests using a significance level of 0.05. Statistical analyses were performed using GraphPad Prism version 9. Bootstrapping was performed using the dabest_v03.1 Python package^66^.

### PET imaging

PET imaging was performed as described previously in ref. ^67^ using an Inveon preclinical PET/CT system (Siemens). After starting the PET scan, 7–8 MBq of [^18^F]FDG in 50–100 ml saline was injected per mouse and emission data were acquired for 45 min. The CT data were used for attenuation correction of the PET data and the CT image was used for image co-registration and BAT area was localized based in co-registered images. The [^18^F]FDG signal in the BAT was normalized by the total [^18^F]FDG signal in the mouse, as the whole mouse was in the field of view of the PET scanner.

### Hepatic SNA recording

Experiments were conducted at the University of Iowa as previously described^17^. Male and female mice were shipped and acclimatized before the experiment. Mice were anaesthetized by i.p. injection of a ketamine–xylazine mixture during the procedure. Baseline measurements of liver SNA were obtained during 5–10 min before i.v. injection of vehicle or CNO (1 mg per kg body weight, i.v.) and recorded for the following hour.

### Whole-brain immunostaining

All samples were processed following a similar protocol as described in ref. ^21^ with minor modifications.

#### Sample pretreatment

Fixed brains were pretreated before immunostaining as follows: dehydration of samples by a 1-h incubation with serial dilutions of 20%, 40%, 60%, 80% and 100% methanol/H_2_O, followed by a 1-h incubation in 100% methanol at 4 °C and an overnight incubation in 66% dichloromethane (DCM)/methanol. Samples were incubated again in 100% methanol at 4 °C and bleached by overnight incubation in 5% H_2_O_2_ in methanol at 4 °C. Next, samples were rehydrated by serial incubations (1 h) of 80%, 60%, 40% and 20% methanol and PBS and washed with 0.2% Triton X-100/PBS.

#### Immunostaining

Pretreated samples were incubated in 0.2% Triton X-100/20% DMSO/0.3 M glycine in PBS at 37 °C for 2 d, then blocked in 0.2% Triton X-100/10% DMSO/6% donkey serum/PBS at 37 °C for 2 d. Samples were incubated with rabbit-anti-Fos (1:1,000 dilution; Cell Signaling, 2250) in 0.2% Triton X-100/5% DMSO/3% donkey serum/PBS for 7 d at 37 °C, followed by 4× washes with 0.2% Triton X-100/PBS along 48 h. Finally, samples were incubated with donkey anti-rabbit Alexa Fluor 647 (1:500 dilution; Invitrogen/Thermo, A31573) in 0.2% Triton X-100/5% DMSO/3% donkey serum/PBS for 7 d at 37 °C and washed again in 0.2% Triton X-100/PBS for 48 h.

#### Brain clearing

The protocol was adapted from ref. ^68^. Samples were dehydrated with serial incubations (1 h) in 20%, 40%, 60%, 80% and 100% methanol/H_2_O, followed by overnight incubation in 100% methanol. Subsequently, samples were incubated in 66% DCM/methanol for 5 h and incubated twice in 100% DCM for 30 min. Samples were stored in ethyl cinnamate (Eci, Sigma, 112372) until imaging.

#### Light sheet fluorescence microscopy imaging

The cleared samples were imaged with LaVision Bio Tec Ultramicroscope II with Inspector Pro (V.5.0.222.0) software, using Eci as imaging medium. Whole-brain scans were taken at 0.68× from ventral to dorsal orientation along 5,200 µm, capturing 1,300 planes with 4-µm steps. Each sample was scanned using 488-nm and 630-nm wavelengths to image the sample’s physical boundaries and the specific Fos signal, respectively. Scans were taken using same laser intensity and settings for all samples.

### Unbiased whole-brain Fos distribution analysis

#### Co-registration

The direct co-registration of the whole-brain 630-nm images to the Allen Brain 25-μm reference mouse brain atlas (https://download.alleninstitute.org/informatics-archive/october-2014/annotation/atlasVolume.zip) was not optimal in several samples after visual inspection of the alignment of the following landmarks: (1) the anatomical surfaces of the hypothalamic area, (2) the alignment of the third and lateral ventricles and (3) the general structure of the hindbrain. An adapted workflow from our previous protocol described in ref. ^21^ was used. Whole-brain 630-nm images were co-registered to the whole-brain 488-nm image scans of the same mouse, and then themselves co-registered to the Allen brain atlas. A 12-parameter affine mutual information schema for the in-mouse co-registrations of 630-nm and 488-nm images was used, followed by a different 12-parameter affine mutual information schema for the co-registration into the atlas. Co-registrations were done using Vinci version 5.06.0 (https://vinci.sf.mpg.de/). The best co-registration was selected as the master brain, and the rest of the samples were subsequently co-registered to the master brain. Visual inspection of the co-registered samples was used to exclude samples (one sample from AgRP-Gq, POMC-Gi and AgRP-Gq, POMC-Gi groups) that did not fulfil two of the three criteria mentioned above.

#### Processing of Fos images

Whole-brain Fos data were stored in 3D datasets (voxel size = 25 µm × 25 µm × 25 µm, dimensions of 440 × 320 × 520). For local statistical testing, the local density of Fos^+^ cells were determined. To this end, Fos^+^ cells were identified by application of the following procedures: (1) identification of potential Fos-positive voxels by the requirement of 20% increase in voxel value compared to the 26 surrounding voxels. To avoid artefacts at the edge of the brain tissue, only voxels with all surrounding voxels being located within the brain tissue are taken into account. (2) Identification of clusters of voxels rated as Fos^+^, which would affect eight voxels at a maximum. Only clusters consisting of one to eight voxels were counted as one Fos^+^ cell, while other clusters were regarded as artefacts. (3) Local density (cells per volume) of Fos^+^ cells was calculated by counting the number of positive cells in volumes consisting of 20 × 20 × 20 voxels (0.125 µl) by application of a uniform 20 × 20 × 20 kernel. The resulting image is in units of cells per microlitre. To identify regions with differences in Fos expression between the groups, a voxelwise *t*-test was performed and results were displayed as images of the *P* values. The calculations were performed using our own codes written in IDL (version 8.5.1) and c (gcc Ubuntu 7.5.0-3).

### Hindbrain single-nucleus sequencing

Mice were euthanized by cervical dislocation 1 h after CNO or vehicle injection in the absence of food, and a portion of the hindbrain containing the AP and NTS was dissected as shown in ref. ^38^. Samples from the same experimental condition were pooled together (*n* = 2 mice per group)

#### Nuclei sorting protocol

Sorting by flow cytometry was performed using a BD FACSAria IIIu Influx cell sorter (BD Biosciences). The gating was set according to size and granularity using FSC and SSC to capture singlets and remove debris, and fluorescence was set at 647 nm and 670 nm to detect DraQ5 staining. Each sample was sorted into separate tubes, each with a total of 20,000 particles per 40 µl.

#### Single-nucleus RNA sequencing

Sequencing libraries for the four single-nucleus suspension samples were generated using 10x Genomics Chromium Single-Cell 3′ Reagent kit according to the standardized protocol. Paired-end sequencing was performed using an Illumina NovaSeq 6000 (read 1: 28 bp; read 2: 91 bp). Library preparation and sequencing was performed by the Cologne Center for Genomics.

### Hindbrain single-nucleus RNA-sequencing analysis

Two single-nucleus samples were obtained for each condition. Raw sequence reads were mapped and genes counted based on the GRCm38 reference genome assembly (mm10) using 10x Genomics Cell Ranger (v7.0.0)^69^. The filtered raw count matrices obtained from Cell Ranger were then further processed using the Seurat R package (v4.3.0)^70^. Cells with fewer than 800 unique molecular identifiers, fewer than 500 unique genes or a rate of mitochondrial RNA above 10% were removed. We used scDblFinder (https://github.com/chris-mcginnis-ucsf/DoubletFinder/)^71^ to detect doublets in the data, assuming a doublet rate of 0.02. Highly variable genes (HVGs) were detected using Seurat’s ‘SelectIntegrationFeatures’ function, which allows selection of the consensus HVGs across all samples. IEGs and other possibly confounding genes were removed from the HVGs. Next, scvi-tools (version 0.16.4) was used to train a scvi model (‘scvi.model.SCVI’) adjusting for sample and using the top 2,000 HVGs^72^. The low-dimensional scvi embedding was used for all further analyses. The scvi model was trained for 80 epochs and with an increased number of latent dimensions to better represent the neuronal heterogeneity^73^. Clustering was conducted using Louvain community detection on the *k*-nearest-neighbours graph as implemented in Seurat. For each cluster, marker genes were detected using the standard Wilcoxon test approach implemented in Seurat′s ‘FindMarkers’ function. Major cell-type annotations for each cluster were obtained using signatures obtained from our previous work^73^ and using the function ‘AddModuleScore’ from Seurat. Neuronal clusters were further labelled by concatenating the two strongest marker genes and the cluster number. To detect differentially expressed genes between the AgRP-Gq;POMC-Gi and control groups within each cluster, we utilized a linear model accounting for sample-level effects as implemented in the nebula R package (version 1.2.1)^39^. The results were corrected for multiple testing using the Holm–Bonferroni method. To determine which clusters were potentially activated by AgRP-Gq;POMC-Gi stimulation, we focused on a set of 10 IEGs (*Fos*, *Fosl2*, *Homer1*, *Nr4a3*, *Nr4a1*, *Gem*, *Jun*, *Junb*, *Btg1* and *1700016P03Rik*) that we identified as good indicators of potential neuronal activation from a list of 140 IEGs^73,74^. The *P* values of these 10 relevant IEGs were combined for each of the 136 neuronal clusters, only including IEGs expressed in at least 5% of cells and significantly different (*P* < 0.05) in the cluster, using Fisher’s method. We then used the negative logarithm of the chi-squared test statistic as a score to indicate neuronal activation, setting clusters with no significantly changing IEGs to 0. If the average log_2_ fold change of significant IEGs was negative, we changed the sign of the score, thereby indicating potential inhibition. Due to the weak overall IEG signal, we did not use the adjusted *P* values for calculation of this score, but note that this approach potentially retains false positives and hence further validation of activated clusters is required. Generation of single-nucleus figure plots was conducted with Seurat and the ggplot2 R package (version 3.4.2).

### Statistics

Statistical analyses were performed using GraphPad Prism version 9. Significance was accepted when the *P* value was lower than 0.05. Data distribution was assumed to be normal, but this was not formally tested in all the experiments. One-way ANOVA was performed when only one variable (genotype) was being compared. Two-way ANOVA was performed followed by a post hoc test when two variables were being compared. The number of replicates (*n*) are indicated in the figure legends. No statistical methods were used a priori to predetermine sample sizes, although group sizes used were similar to those commonly applied in mouse studies.

### Reporting summary

Further information on research design is available in the Nature Portfolio Reporting Summary linked to this article.

### Supplementary information

Supplementary Information Supplementary Figs. 1–5

Reporting Summary

### Source data

Source Data Fig. 1 Raw data, *n* numbers and statistical summary.

Source Data Fig. 2 Raw data, *n* numbers and statistical summary.

Source Data Fig. 3 Raw data, *n* numbers and statistical summary.

Source Data Fig. 4 Raw data, *n* numbers and statistical summary.

Source Data Fig. 5 Raw data, *n* numbers and statistical summary.

Source Data Fig. 6 Raw data, *n* numbers and statistical summary.

Source Data Fig. 7 Raw data, *n* numbers and statistical summary.

Source Data Extended Data Fig./Table 1 Raw data, *n* numbers and statistical summary.

Source Data Extended Data Fig./Table 2 Raw data, *n* numbers and statistical summary.

Source Data Extended Data Fig./Table 3 Raw data, *n* numbers and statistical summary.

Source Data Extended Data Fig./Table 4 Raw data, *n* numbers and statistical summary.

Source Data Extended Data Fig./Table 5 Raw data, *n* numbers and statistical summary.

Source Data Extended Data Fig./Table 6 Raw data, *n* numbers and statistical summary.

Source Data Extended Data Fig./Table 7 Raw data, *n* numbers and statistical summary.

Source Data Extended Data Fig./Table 8 Raw data, *n* numbers and statistical summary.

Source Data Extended Data Fig./Table 9 Raw data, *n* numbers and statistical summary.

## Data Availability

Raw data were generated from aligned 3D whole-brain scans and data analysis derived from the unbiased whole-brain Fos distribution analysis are available on request to J.C.B. The single-nucleus sequencing data are available from the Gene Expression Omnibus under accession number GSE248391. Source data are provided with this paper.

## Code availability

Code generated for the processing of Fos images obtained by whole-brain Fos immunostaining is available on request. Code for the processing and analysis of the single-nucleus sequencing data, as well as code for generating the related figures, is available from https://github.com/lsteuernagel/AgrpPomc_NTS/.
