# Supplementary material for: Reciprocal activity of AgRP and POMC neurons governs coordinated control of feeding and metabolism
Source: Nat Metab. 2024 Feb 20;6(3):473–93. doi: 10.1038/s42255-024-00987-z (PMC10963273; doi:10.1038/s42255-024-00987-z)
Supplement: Supplementary file 1 — Supplementary Figs. 1–5 [file 42255_2024_987_MOESM1_ESM.pdf]

nature metabolism

Article

https://doi.org/10.1038/s42255-024-00987-z

Reciprocal activity of AgRP and POMC neurons governs coordinated control of feeding and metabolism

In the format provided by the authors and unedited

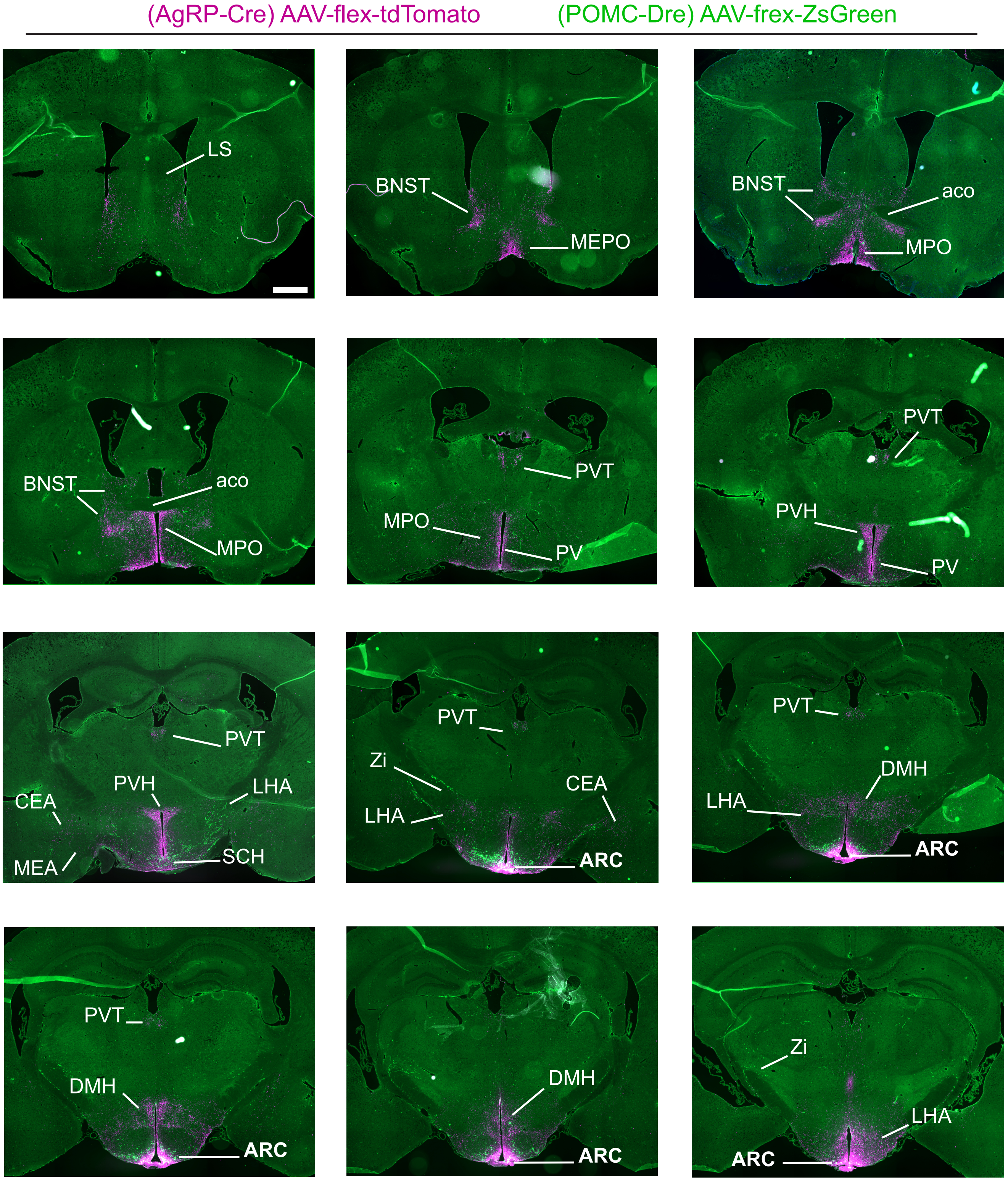

LS: Lateral septum
BNST: Bed nuclei of the stria terminalis
MEPO: Median preoptic nucleus
aco: anterior commissure
MPO: Medial preoptic nucleus
PVT: Paraventricular nucleus of the thalamus
PV: Periventricular nucleus
PVH: Periventricular nucleus of the hypothalamus
LHA: Lateral hypothalamus
SCH: Suprachiasmatic nucleus
CEA: Central amygdala area
MEA: Medial amygdala area
ZI: zona incerta
ARC: Arcuate nucleus of the hypothalamus
DMH: dorsomedial hypothalamic nucleus

Supplementary Figure 1 (1/2). De Solis AJ *et al.*

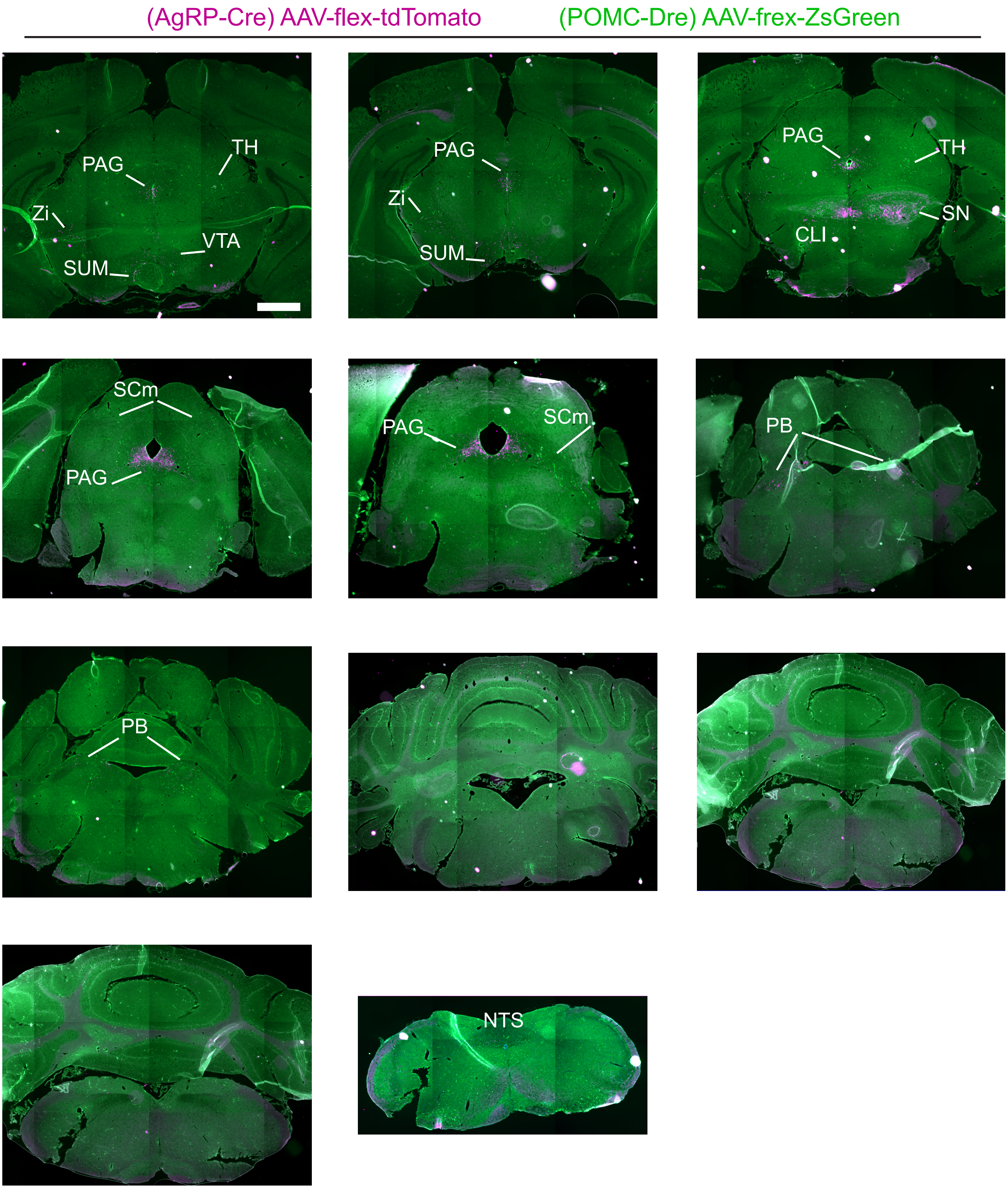

ZI: Zona incerta: Xona incerta
VTA: Ventral tagmenetal area
SUM: Suprammamiliary nucleus
TH: Thalamus
CLI: Raphe,central linear nucleus

SN: Substancia nigra
PAG: Periaqueductal grey
SCm: Superior culliculus, motor related
PB: Parabranqial nucleus
NTS: Nucleus of the tractus solitarius

Supplementary Figure 1 (2/2). De Solis AJ et al

Supplementary Figure 1. Whole brain distribution of overlapping AgRP and POMC axonal projections. Representative coronal images show the distribution of $AgRP^{+}$ (magenta) and $POMC^{+}$ (green) axonal projections after bilateral injection of AAV-flex-tdTomato and AAV-frex-ZsGreen into the ARC area of AgRP-Gq;POMC-Gi mice (n = 2). Scale bar = 200 μm.

Donwstream area		AgRP-tdTomato projections	POMC-ZsGreen projections
LS	Lateral septum	++	+
BNST anterior	Bed nucleus of the striata terminals, anterior area	+++	+
BNST medial	Bed nucleus of the striata terminals, medial area	+++	++
BNST posterior	Bed nucleus of the striata terminals, posterior area	+++	+
NDB	Diagonal band nucleus	++	--
MS	Medial septum nucleus	+	--
LPO	Lateral preoptic area	--	--
ACB medial	Nucleus accumbens	++	+
MEPO	Median preoptic nucleus	++	+
MPO	Medial preoptic area	+++	++
PAL / MA	Pallidum, magnocellular nucleus	+	--
PVT anterior	Paraventricular nucleus of the thalamus, anterior area	++	+
PVT medial	Paraventricular nucleus of the thalamus, medial area	+	+
PVT posterior	Paraventricular nucleus of the thalamus, posterior area	--	--
AHN	Anterior nucleus of the hypothalamus	+	++
PVH	Paraventricular nucleus of the hypothalamus	++++	++
LHA	Lateral hypothalamic area	++	+++
AAA	Anterior hypothalamic nucleus	+	+
CEA	Central amygdalar nucleus	+	+
MEA	Medial amygdalar nucleus	+	--
PVi	Periventricular hypothalamic nucleus	++	--
VMH	Ventromedial hypothalamic nucleus	--	--
TU	Tuberal nucleus	++	++
DMH	Dorsomedial hypothalamic nucleus	+++	+
Zi	Zona incerta	+	--
Zi, posterior	Zona incerta, posterior part	--	+
PVp	Periventricular hypothalamic nucleus	+++	+
PH	Posterior hypothalamic nucleus	+++	+
ME	Median eminence	+	+
PAG, anterior	Periaqueductal grey, anterior area	++	+
PAG, posterior	Periaqueductal grey, posterior area	+++	+
SC	Superior colliculus, motor related area	+	++
VTA	Ventral tegmental area	++	--
APN	Anterior pretectal nucleis (Thalamus region)	--	++
CLI	Central linear nucleus raphe	++	--
SNc	Substancia nigra, campact part	--	+
PB	Parabranquial nucleus	++	+
RM	Nucleus of the raphe magnus	--	+
PCG	Pontine central grey	+	+
GRN	Gigantocellular reticular nucleus	--	+
NTS	Nucleus of the solitary tract	+	+
AP	Area postrema	--	--

Supplementary Figure 2. De Solis AJ *et al.*

Supplementary Figure 2. List of brain areas receiving AgRP and POMC axonal projections. Quantification of intensity was performed by direct visual inspection for each channel independently (*n* = 2 mice). Area nomenclature is based on Allen mouse brain atlas.

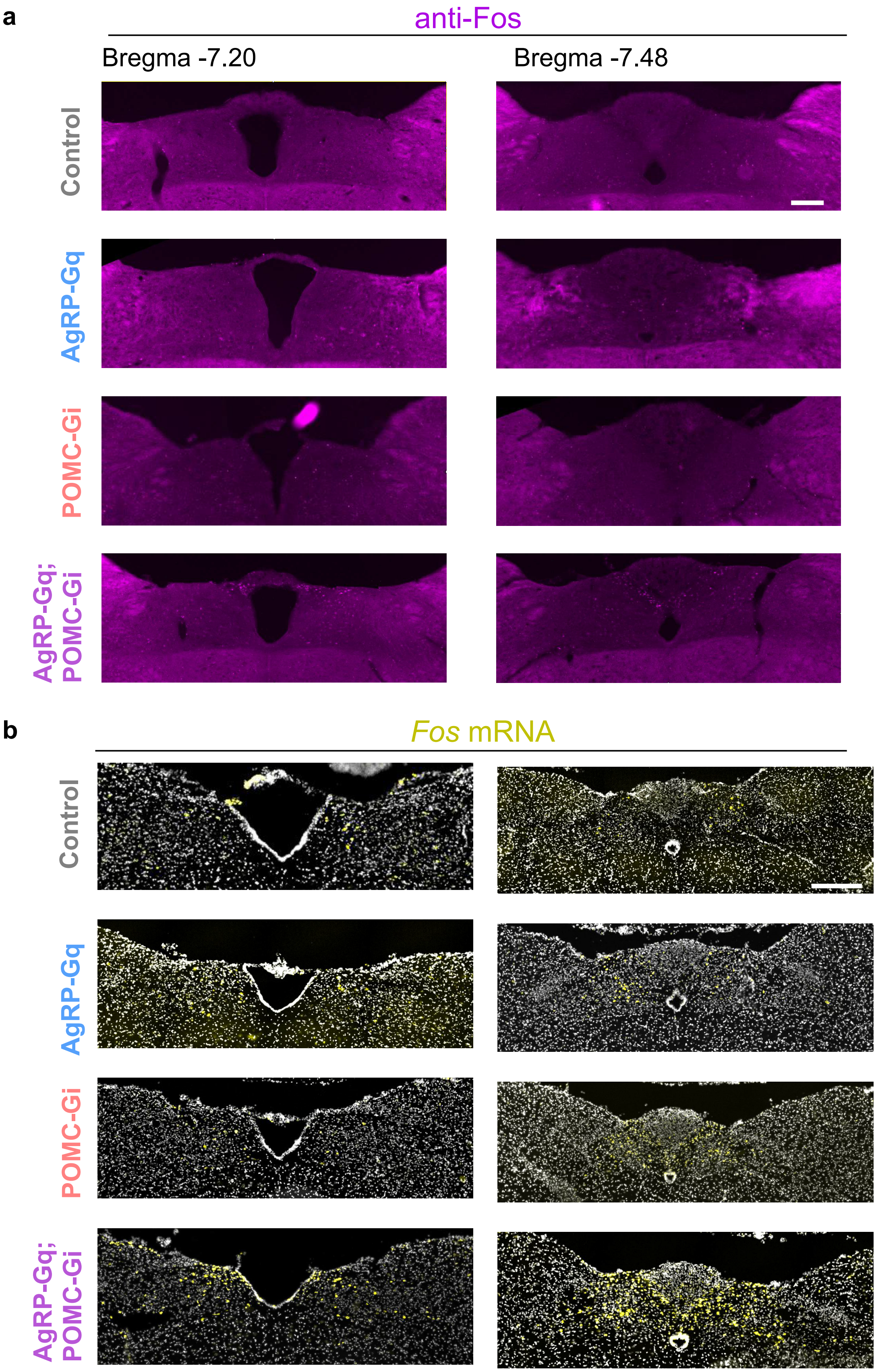

Supplementary Figure 3. De Solis AJ *et al.*

Supplementary Figure 3. Fos distribution in the NTS area after chemogenetic intervention. **a,** Representative pictures of Fos protein expression in medial (bregma -7.20) and posterior (bregma -7.48) NTS area. **b,** Representative images of Fos mRNA expression in medial and posterior NTS area from mice of the four experimental groups treated with CNO. Quantification of Fos protein showed in **Fig. 3h** and quantification of *Fos*$^{+}$ mRNA cells showed in **Fig. 3i**.

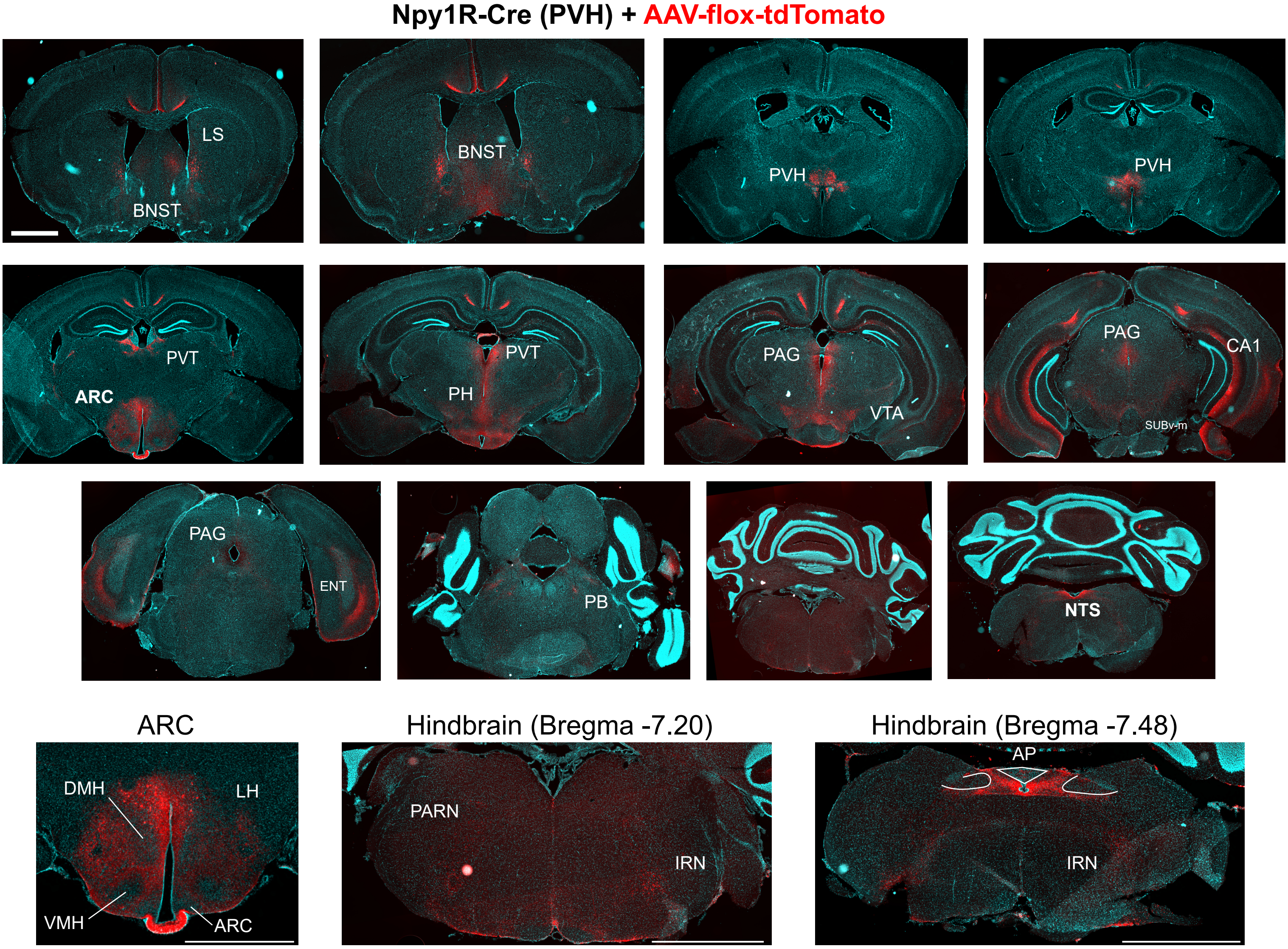

ARC: Arcuate nucleus of the hypothalamus

AP: Area postrema

BNST: Bed nucleus of the stria terminales

DMH: Dorsomedial nucleus of the hypothalamus

ENT: Enthorinal area

IRN: Intermediate reticular nucleus

LH: Lateral hypothalamus

LS: Lateral septum

ME: Mediam eminence

NTS: Nucleus of the tractus solitarius

PAG: Periaqueductal gray

PARN: Parvicellular reticular nucleus

PBN: Parabraquial nucleus

PVH: Paraventricular nucleus of the hypothalamus

PVT: Paraventricular nucleus of the thalamus

SUBv-m: Subcubiculum ventral, molecular layer

VHM: Ventromedial nucleus of the hypothalamus

VTA: Ventral tegmental area

Supplementary Fig. 4. De Solis AJ *et al.*

Supplementary Figure 4. Npy1R^{PVH} axonal projections into hindbrain region. Representative images of stained axonal projections (red) from Npy1R^{PVH}–TdTomato^{+} neurons across the whole brain. Images selected from one mouse within 3 biological replicates. Lower panels showed the distribution of Npy1R-Tomato^{+} fibers around the main nuclei in the ventral hypothalamus (left) and in the hindbrain at the level of medial NTS area (bregma – 7.20) and posterior NTS/AP area (bregma -7.48), where the medial part of the posterior NTS receives extensive Npy1R^{PVH} projections, while AP and DMV areas receive less axonal projections. Scale bar = 1500 μm.

RNAscope probes	Reference	Dilution	Accession number
AgRP	#400711	1:2	NM_001271806.1
POMC	#314081	1:4	NM_008895.3
Fos	#316921	1:1	NM_010234.2
ZsGreen	#461251	1:1	JQ071441.1
eGFP	#400281	1:1	NM_U55763.1
Cre	#312281	1:1	KC845567.1
Dre	#442641	1:1	N/A
MC4R	#319181	1:1	NM_016977.4
Npy1R	#427021	1:1	NM_010934.4
Glp1R	#418851	1:1	NM_021332.1
Pdyn	#318771	1:1	NM_018863.3
Slc17a6	#319171	1:1	NM_080853.3
Slc32a1	#319191	1:1	NM_009508.2
Th	#317621	1:1	NM_009377.1
Dbh	#407851	1:1	NM_138942.3

Type of antibodies	Company	Reference	Dilution
Histology. Primary antibodies			
rabbit anti-ZsGreen	Takara Bio Clontech	632474	1:100
rat anti-mCherry (for tdTomato)	Thermo Fisher Scientific	M11217	1:1,000
goat anti-tdtomato	SiCGen	#AB8181	1:500
rabbit c-Fos	Cell signalling	#2250	1:1000
Histology Secondary atibodies			
donkey anti-rabbit Alexa Fluor 488	Thermo Fisher	A21206	1:500
donkey anti-rat Alexa Fluor 594	Jackson ImmunoResearch	712-585-153	1:500
goat anti-rabbit Alexa Fluor 594	Thermo Fisher Scientific	A11012	1:500
Whole brain immunohistology			
rabbit c-Fos	Cell signalling	#2250	1:1000
donkey-anti-rabbit-Alexa647	Invitrogen/Thermo	A31573	1:1000

Other reagents	Company	Reference
Taqman qPCR probes		
Hprt	Thermo Fisher Scientific	Mm01545399_m1
G6pc	Thermo Fisher Scientific	Mm00839363_m1
Pck1	Thermo Fisher Scientific	Mm01247058_m1
Addgene virus stocks		
AAV1-flex-TdTomato	Addgene	# 28306
AAV1-CAG-Frex-ZsGreen	Addgene	#52521
AAV8-hSyn-DIO-hM4DGi-mCherry	Addgene	# 44362

Supplementary Figure 5. De Solis *et al.* .
