# Supplementary material for: Reciprocal activity of AgRP and POMC neurons governs coordinated control of feeding and metabolism
Source: Nat Metab. 2024 Feb 20;6(3):473–93. doi: 10.1038/s42255-024-00987-z (PMC10963273; doi:10.1038/s42255-024-00987-z)
Supplement: Supplementary file 2 — Reporting Summary [file 42255_2024_987_MOESM2_ESM.pdf]

nature portfolio

Corresponding author(s): Jens C. Brüning

Last updated by author(s): Dec 12, 2023

Reporting Summary

Nature Portfolio wishes to improve the reproducibility of the work that we publish. This form provides structure for consistency and transparency in reporting. For further information on Nature Portfolio policies, see our Editorial Policies and the Editorial Policy Checklist.

Statistics

For all statistical analyses, confirm that the following items are present in the figure legend, table legend, main text, or Methods section.

n/a	Confirmed	
☐	☒	The exact sample size (*n*) for each experimental group/condition, given as a discrete number and unit of measurement
☐	☒	A statement on whether measurements were taken from distinct samples or whether the same sample was measured repeatedly
☐	☒	The statistical test(s) used AND whether they are one- or two-sided<br>*Only common tests should be described solely by name; describe more complex techniques in the Methods section.*
☒	☐	A description of all covariates tested
☐	☒	A description of any assumptions or corrections, such as tests of normality and adjustment for multiple comparisons
☐	☒	A full description of the statistical parameters including central tendency (e.g. means) or other basic estimates (e.g. regression coefficient) AND variation (e.g. standard deviation) or associated estimates of uncertainty (e.g. confidence intervals)
☐	☒	For null hypothesis testing, the test statistic (e.g. *F*, *t*, *r*) with confidence intervals, effect sizes, degrees of freedom and *P* value noted<br>*Give P values as exact values whenever suitable.*
☒	☐	For Bayesian analysis, information on the choice of priors and Markov chain Monte Carlo settings
☒	☐	For hierarchical and complex designs, identification of the appropriate level for tests and full reporting of outcomes
☒	☐	Estimates of effect sizes (e.g. Cohen's *d*, Pearson's *r*), indicating how they were calculated

Our web collection on statistics for biologists contains articles on many of the points above.

Software and code

Policy information about availability of computer code

Data collection	Indirect calorimetry: TSE PhenoMaster versions 6.2.5 and above<br>CT imaging: IVIS SpectrumCT scanner and LivingImage Software (version 4.3.1)<br>qPCR analysis: QuantStudio Real-Time PCR software (version 1.7.1)<br>RNA concentration was measured with software ND-1000 (version 3.8.1)<br>Image acquisition: Leica Sp8, Zeiss ImagerM2, LaVision Ultramicroscope II.<br>PET/CT scan: Inveon preclinical<br>Electrophysiology recordings: PatchMaster HEKA<br>snRNAseq: Illumina NovaSeq 6000.
Data analysis	We used Graphpad Prism (Version 9 and 10) for data and statistical analysis.<br>Data analysis for electrophysiological recordings was performed with Spike2 (CED), Igor Pro 6 (Wavemetrics) and Graphpad Prism (version 5.0c)<br>Data analysis for co-registration of 3D whole brain scans were performed in Vinci (version 5.060)<br>Data analysis of Fos distribution in 3D data set of whole brains were perfomerd using own code, developed in IDL (Version 8.5.1) and c (gcc Ubuntu 7.5.0-3).<br><br>The code for the single nucleus data analysis can be found in the repository: https://github.com/lsteuernagel/AgrpPomc_NTS .<br>Raw data were processed using Cellranger (10x Genomics) version:7.0.0.<br>Postprocessing and analysis were conducted using R (version: 4.2.0) and python (version: 3.10.6)<br><br>The following R packages were used:<br>ggh4x - version:0.2.4<br>nebula - version:1.2.1

scDblFinder - version:1.12.0
SingleCellExperiment - version:1.20.0
SummarizedExperiment - version:1.28.0
Biobase - version:2.58.0
GenomicRanges - version:1.50.2
GenomeInfoDb - version:1.34.4
IRanges - version:2.32.0
S4Vectors - version:0.36.1
BiocGenerics - version:0.44.0
MatrixGenerics - version:1.10.0
matrixStats - version:0.63.0-9003
Matrix - version:1.5-3
scUtils - version:0.0.1
magrittr - version:2.0.3
SeuratObject - version:4.1.3
Seurat - version:4.3.0
forcats - version:0.5.2
stringr - version:1.5.0
dplyr - version:1.1.0
purrr - version:1.0.1
readr - version:2.1.3
tidyr - version:1.3.0
tibble - version:3.1.8
tidyverse - version:1.3.2
ggpubr - version:0.6.0
ggplot2 - version:3.4.2

The following python packages were used:
scanpy - version 1.9.1
scvi - version 0.16.4
anndata - version 0.8.0

For manuscripts utilizing custom algorithms or software that are central to the research but not yet described in published literature, software must be made available to editors and reviewers. We strongly encourage code deposition in a community repository (e.g. GitHub). See the Nature Portfolio guidelines for submitting code & software for further information.

Data

Policy information about availability of data

All manuscripts must include a data availability statement. This statement should provide the following information, where applicable:

- Accession codes, unique identifiers, or web links for publicly available datasets
- A description of any restrictions on data availability
- For clinical datasets or third party data, please ensure that the statement adheres to our policy

Raw data were generated from aligned 3D whole brain scans, and the code and data analysis derived from the processing of the Fos images obtained by whole brain immunostaining are available on request to the Corresponding Author.

The single-nucleus sequencing data is available from GEO under the accession number: GSE248391

Code for the processing and analysis of the single-nucleus sequencing data, as well as code for generating the related figures is available from https://github.com/lsteuernagel/AgrpPomc_NTS .

Raw data collected from experiments, the summary of statistical analysis, including exact P values and the n number of biological replicates segregated by sex are provided in the Source Data files.

Field-specific reporting

Please select the one below that is the best fit for your research. If you are not sure, read the appropriate sections before making your selection.

☒ Life sciences ☐ Behavioural & social sciences ☐ Ecological, evolutionary & environmental sciences

For a reference copy of the document with all sections, see nature.com/documents/nr-reporting-summary-flat.pdf

Life sciences study design

All studies must disclose on these points even when the disclosure is negative.

Sample size	We performed a sample size calculation required by the Ethical authorities, although no specific sample size calculation was performed for each experiment. The sample size of each experiment was determined by the common practice with rodent experiments, based on previous experiments (Gaziano I. 2022, Engström Ruud L. 2020, Chen W. 2023).<br>For RNAscope validation we aimed for a mimimun of 3 mice with 2-4 sections containing both hemisections of the area of interest.

Data exclusions	Mice that presented a deviation more than twice the SEM from the average body weight of their sex and experimental group were removed from the experiments.<br>Data were excluded only due to technical errors that occurred in during Phenomaster recordings (i.e. unexpected food intake increases >2g/20 min) or due to AAV stereotaxic injection outside of PVH area.
Replication	All experiments used litter mate mice from at least 3-4 different cohorts to ensure enough genetic and physiological variability throughout the experiments. No significant differences were observed between cohorts in the data analysis.<br>All attempts for replicates were successful.
Randomization	Mice were randomly distributed before receiving experimental treatments, always considering balanced sex and body weight distribution in all experimental groups. Chemogenetic experiments were designed in cross-experimental design, ensuring that each mouse received both the chemogenetic actuator (clozapine-N_oxide, CNO) and the vehicle (saline solution, 3% DMSO)
Blinding	Data collection from physiological experiments with mice and data analysis were carried in blind conditions for genotypes.<br>Image acquisition and image analysis were performed in blind conditions.<br>Data analysis of sequencing data (e.g., single-cell data processing and differential expression analysis) was not carried out in a blinded fashion, due to the common workflows requiring availability of metadata.

Reporting for specific materials, systems and methods

We require information from authors about some types of materials, experimental systems and methods used in many studies. Here, indicate whether each material, system or method listed is relevant to your study. If you are not sure if a list item applies to your research, read the appropriate section before selecting a response.

Materials & experimental systems

n/a	Involved in the study
☐	☒ Antibodies
☒	☐ Eukaryotic cell lines
☒	☐ Palaeontology and archaeology
☐	☒ Animals and other organisms
☒	☐ Human research participants
☒	☐ Clinical data
☒	☐ Dual use research of concern

Methods

n/a	Involved in the study
☒	☐ ChIP-seq
☐	☒ Flow cytometry
☒	☐ MRI-based neuroimaging

Antibodies

Antibodies used	The list of all antibodies used in this study can be found in the Supplementary Figure 5.<br><br>rabbit anti-ZsGreen, Takara Bio Clontech, #632474<br>rat anti-mCherry, Thermo Fisher Scientific, #M11217<br>goat anti-tdtomato, SiCGen #AB8181<br>rabbit anti-c-Fos, Cell signalling #2250<br>donkey anti-rabbit Alexa Fluor 488, Thermo Fisher Scientific, #A21206<br>donkey anti-rat Alexa Fluor 594, Jackson ImmunoResearch, #712-585-153<br>goat anti-rabbit Alexa Fluor 594, Thermo Fisher Scientific, #A11012<br>donkey-anti-rabbit-Alexa647 Invitrogen/Thermo #A31573
Validation	All antibodies were validated by the experience of the Histological analysis unit and other investigators at the Max Planck Institute for Metabolism Research. Additionally, antibodies were validated by the manufacturer company:<br><br>Rabbit anti-ZsGreen (Takara Bio Clontech), cited 7 times in webpage. https://www.takarabio.com/documents/Certificate%20of%20Analysis/632474/632474-042612.pdf<br>rat anti-mCherry (Thermo Fisher Scientific), cited by 128 references. https://www.thermofisher.com/antibody/product/mCherry-Antibody-clone-16D7-Monoclonal/M11217<br>goat anti-tdtomato (SiCGen), cited 7 times at the company's webpage. https://zhscience.com/Uploads/file/20230327/AB7358-200.pdf<br>rabbit c-Fos (Cell signalling), cited by >500 publications. https://www.cellsignal.com/products/primary-antibodies/c-fos-9f6-rabbit-mab/2250<br>donkey anti-rabbit Alexa Fluor 488 (Thermo Fisher), cited in >4000 references. https://www.thermofisher.com/antibody/product/Donkey-anti-Rabbit-IgG-H-L-Highly-Cross-Adsorbed-Secondary-Antibody-Polyclonal/A-21206<br>donkey anti-rat Alexa Fluor 594 (Jackson ImmunoResearch), cited by 29 references. https://www.jacksonimmuno.com/catalog/products/712-585-153<br>goat anti-rabbit Alexa Fluor 594 (Thermo Fisher Scientific), cited in >1900 references. https://www.thermofisher.com/antibody/product/Goat-anti-Rabbit-IgG-H-L-Cross-Adsorbed-Secondary-Antibody-Polyclonal/A-11012<br>Donkey anti-rabbit-Alexa 647 (Thermo Fisher) with > 1500 references, https://www.thermofisher.com/antibody/product/Donkey-anti-Mouse-IgG-H-L-Highly-Cross-Adsorbed-Secondary-Antibody-Polyclonal/A-31571

Animals and other organisms

Policy information about studies involving animals; ARRIVE guidelines recommended for reporting animal research

Laboratory animals	Mice were group housed (3-5 animals per cage) in a controlled environment regarding to humidity and temperature (22-24°C) on a 12 h light/12 h dark cycle. Mice had ad libitum access to water and to a standard rodent chow diet (CD) (ssniff®, V1154-704), containing 57% of calories from carbohydrates, 34% calories from protein and 9% calories from fat. Additionally, mice were fed ad libitum with high fat diet (HFD, ssniff®, E15742-350) containing 21% calories from carbohydrates, 19% calories from protein and 60% calories from fat. Food was removed for fasting experiments at the indicated times.<br>Mice were maintained in C57BL/6N background. C57BL/6N mice were obtained from Charles River, France. Transgenic mouse lines were described previously: AgRP-IREs-Cre line (Balthasar N, 2004; DOI: 10.1016/j.neuron.2004.06.004), Rosa26-CAG-lox-STOP-lox-hM3DGq-GFP (Madisen L, 2012; doi: 10.1038/nn.3078), POMC-Dre (Biglari N, 2019; DOI: 10.1038/s41593-021-00854-0) and Npy1R-Cre (Padilla SL, 2016; DOI: 10.1038/nn.4274). Line Rosa26-CAG-rox-STOP-rox-hM4DGi-ZsGreen was generated in a similar way as described before (Biglari N, 2019; DOI: 10.1038/s41593-021-00854-0)<br><br>All experiments were performed in adult mice, age 12-20 weeks. All key experiments have been performed in male and female mice separately. Mice were randomly distributed before receiving experimental treatments, always considering balanced sex and body weight distribution in all experimental groups. Chemogenetic experiments were designed in cross-experimental design, ensuring that each mouse received both the chemogenetic actuator and the vehicle. Data analysis was segregated by sex. Actual number of male and female mice used in each experiment is displayed in the figure legends and summarized into the Data source files.<br>in the Source Data files.
Wild animals	The study did not involve wild animals.
Field-collected samples	The study did not involve samples collected from field.
Ethics oversight	All animal procedures were conducted in compliance with protocols approved by local government authorities (Bezirksregierung Köln). Permission for breeding and experiments on mice was issued by the Department for Environment and Consumer Protection-Veterinary Section in Cologne.

Note that full information on the approval of the study protocol must also be provided in the manuscript.

Flow Cytometry

Plots

Confirm that:

☒ The axis labels state the marker and fluorochrome used (e.g. CD4-FITC).

☒ The axis scales are clearly visible. Include numbers along axes only for bottom left plot of group (a 'group' is an analysis of identical markers).

☒ All plots are contour plots with outliers or pseudocolor plots.

☐ A numerical value for number of cells or percentage (with statistics) is provided.

Methodology

Sample preparation	Adult mouse hindbrain area, containing the NTS and AP areas were dissected in HibernateA Medium (ThermoFisher, #A1247501) and snap frozen in liquid nitrogen. Samples were processed following the protocol describes in Dowsett GKC et al. Mol Metabol 2021 (doi.org/10.1016/j.molmet.2021.101240).
Instrument	BD FACSAria IIIu (BD Biosciences)
Software	BD FACSDiva 8.0.1 (BD Biosciences) for data acquisition and FlowJo 10.9.0 for data analysis (BD Biosciences)
Cell population abundance	Cell nuclei were selected and collected for future experimental processing. No specific cell type population was targeted.
Gating strategy	See Extended Data Fig7a: Examplary gating strategy for sorting by flow cytometry: Nuclei determined by side scatter area (SSC-A) vs. forward scatter area (FSC-A). Singlets determined by FSC-A vs. forward scatter width (FSC-W). 2n nuclei determined by DRAQ5 fluorescence (DRAQ5 width vs. DRAQ5 area)

☒ Tick this box to confirm that a figure exemplifying the gating strategy is provided in the Supplementary Information.
